# Chemo-enzymatic modification of poly-*N*-acetyllactosamine (LacNAc) oligomers and *N,N*-diacetyllactosamine (LacDiNAc) based on galactose oxidase treatment

**DOI:** 10.3762/bjoc.8.80

**Published:** 2012-05-09

**Authors:** Christiane E Kupper, Ruben R Rosencrantz, Birgit Henßen, Helena Pelantová, Stephan Thönes, Anna Drozdová, Vladimir Křen, Lothar Elling

**Affiliations:** 1 Laboratory for Biomaterials, Institute for Biotechnology and Helmholtz-Institute for Biomedical Engineering, RWTH Aachen University, Worringer Weg 1, Aachen, 52074, Germany; 2 Institute of Microbiology, Academy of Sciences of the Czech Republic, Videnska 1083, Prague 4, CZ 14220, Czech Republic

**Keywords:** chemo-enzymatic synthesis, galactose oxidase, glycosyltransferase, LacDiNAc, poly-*N*-acetyllactosamine

## Abstract

The importance of glycans in biological systems is highlighted by their various functions in physiological and pathological processes. Many glycan epitopes on glycoproteins and glycolipids are based on *N*-acetyllactosamine units (LacNAc; Galβ1,4GlcNAc) and often present on extended poly-LacNAc glycans ([Galβ1,4GlcNAc]*_n_*). Poly-LacNAc itself has been identified as a binding motif of galectins, an important class of lectins with functions in immune response and tumorigenesis. Therefore, the synthesis of natural and modified poly-LacNAc glycans is of specific interest for binding studies with galectins as well as for studies of their possible therapeutic applications. We present the oxidation by galactose oxidase and subsequent chemical or enzymatic modification of terminal galactose and *N*-acetylgalactosamine residues of poly-*N*-acetyllactosamine (poly-LacNAc) oligomers and *N,N*-diacetyllactosamine (LacDiNAc) by galactose oxidase. Product formation starting from different poly-LacNAc oligomers was characterised and optimised regarding formation of the C6-aldo product. Further modification of the aldehyde containing glycans, either by chemical conversion or enzymatic elongation, was established. Base-catalysed β-elimination, coupling of biotin–hydrazide with subsequent reduction to the corresponding hydrazine linkage, and coupling by reductive amination to an amino-functionalised poly-LacNAc oligomer were performed and the products characterised by LC–MS and NMR analysis. Remarkably, elongation of terminally oxidised poly-LacNAc glycans by β3GlcNAc- and β4Gal-transferase was also successful. In this way, a set of novel, modified poly-LacNAc oligomers containing terminally and/or internally modified galactose residues were obtained, which can be used for binding studies and various other applications.

## Introduction

*N*-Acetyllactosamine (LacNAc; Galβ1,4GlcNAc) structures are important carriers of glycan epitopes, such as ABH or Lewis blood group determinants. Some of these are present on extended poly-*N*-acetyllactosamine glycans (poly-LacNAc; [Galβ1,4GlcNAc]*_n_*) which serve as spacers and additional information carriers [1–5]. Poly-LacNAc itself was identified as a recognition motif of galectins, which are an important class of mammalian lectins [6–7]. Another LacNAc related epitope is LacDiNAc (GalNAcβ1,4GlcNAc), which is especially well-known from parasitic nematodes and trematodes [8–9]. In humans, LacDiNAc-containing glycans trigger the cellular immune response of natural killer cells [10–12]. Scientific interest in the synthesis of naturally occurring and modified LacNAc and poly-LacNAc glycans is high, as they can be used for the detailed investigation of lectin binding modes and their biological function, and as possible diagnostic markers or therapeutic agents [13–16]. Naturally occurring poly-LacNAc glycans and furthermore epitopes thereof, such as fucosylated, sialylated and branched glycans, are widely used in the analysis of galectin–glycan interaction in glycan arrays [7,17–19]. Efforts have especially been made regarding the development and evaluation of galectin inhibitors as therapeutic agents [20–23]. For example, chemically modified mono- and disaccharides as well as thiodigalactosides have been investigated [21,24–27]. Another modification strategy is the use of galactose oxidase, which can be combined with different chemical modifications. Galactose oxidase is a copper-containing enzyme that catalyses the oxidation of the C6-hydroxyl group of nonreducing D-galactose residues [28–29]. Several subsequent modifications, such as site-specific labelling of glycoconjugates with hydrazide or hydroxylamine reagents [30–34], coupling with amino-modified glycan moieties [35], chemical oxidation of the carbonyl to a carboxyl group in the synthesis of immunoreactive neo-glycosaminoglycans [12], and the synthesis of deoxysugars [36], have been reported. The specific properties of galactose oxidase, such as activity and substrate spectrum, have been altered by directed evolution [34,37]. However, although galactose oxidase was discovered decades ago [38–39] and is widely used, the formation of different products during the enzymatic conversion of D-galactose is still not yet completely understood and controllable. The main products of the galactose oxidase reaction are the aldehyde, which occurs as a hydrate in aqueous solution, the corresponding galacturonic acid, and an α,β-unsaturated aldehyde as a dehydrated side product [40–42]. The occurrence of these products seems to vary depending on the synthesis parameters in different applications [12,42–44].

**Scheme 1:**
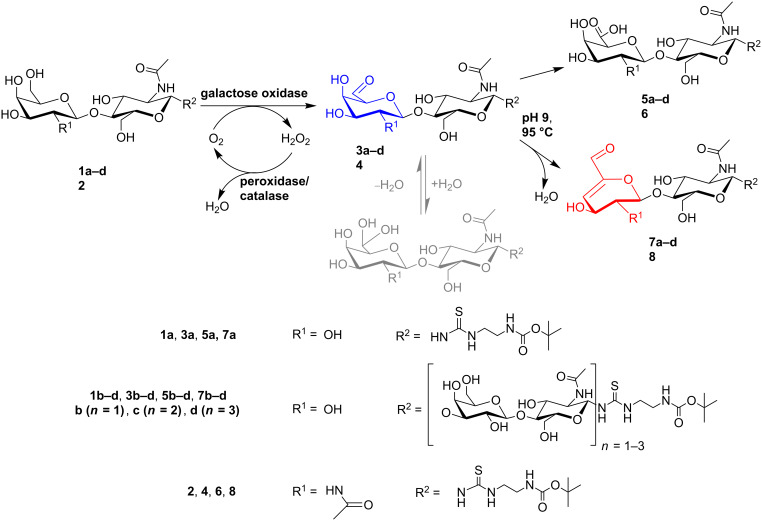
Reaction scheme of terminal galactose in poly-LacNAc-glycans (**1a**–**d**) or GalNAc in LacDiNAc (**2**) by galactose oxidase, to the corresponding 6-aldehyde (**3a**–**d**, **4**). Subsequent further oxidation yields the corresponding galacturonic acids (**5a**–**d**, **6**), and chemical β-elimination the corresponding α,β-unsaturated aldehydes (**7a**–**d**, **8**).

**Scheme 2:**
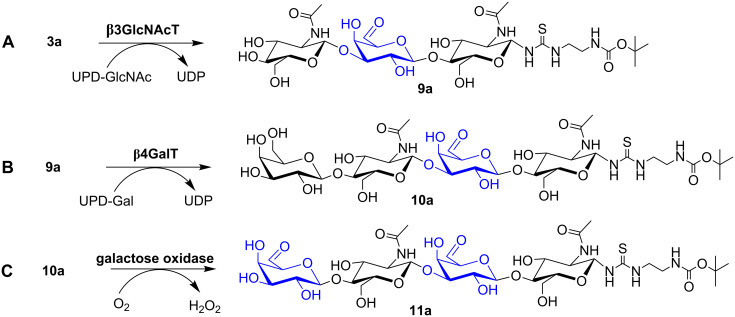
Enzymatic modifications of oxidised poly-LacNAc oligomers. **A:** Elongation of LacNAc-6-aldehyde **3a** by β3-*N*-acetylglucosaminyltransferase (β3GlcNAc-transferase) leads to internally modified trisaccharide **9a**. **B:** The trisaccharide **9a** was further elongated by β4-galactosyltransferase (β4GalT) to yield the di-LacNAc derivative **10a** with an internal C6-oxidised galactose moiety. This reaction proves the synthesis of poly-LacNAc oligomers with internal C6-oxidised galactose units. **C:** Subsequent oxidation of the terminal galactose unit of **10a** showing the potential to produce terminally as well as internally oxidised and modified poly-LacNAc oligomers (**11a**).

**Scheme 3:**
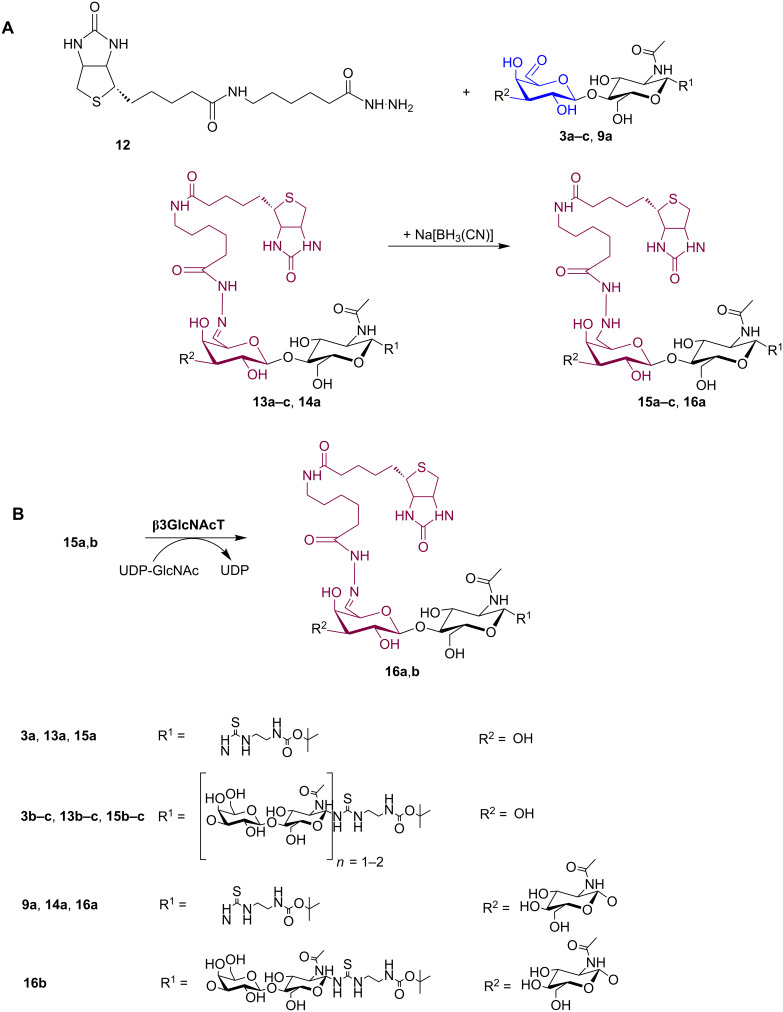
**A:** Labelling of poly-LacNAc oligomers **3a**–**c** and **9a** with biotin hydrazide derivative BACH (**12**) yielding the hydrazone products (**13a**–**c** and **14a**); **13a**–**c** and **14a** were subsequently reduced with Na[BH_3_(CN)] to the corresponding hydrazines (**15a**–**c** and **16a**). **B:** Elongation of biotinylated LacNAc (**15a**) and biotinylated tetrasaccharide (**15b**) by β3-*N*-acetylglucosaminyltransferase (β3GlcNAc-transferase) leads to an internally modified trisaccharide **16a** as the enzymatic elongation of the biotinylated LacNAc **13a** shown in **A**, or pentasaccharide (**16b**), respectively.

**Scheme 4:**
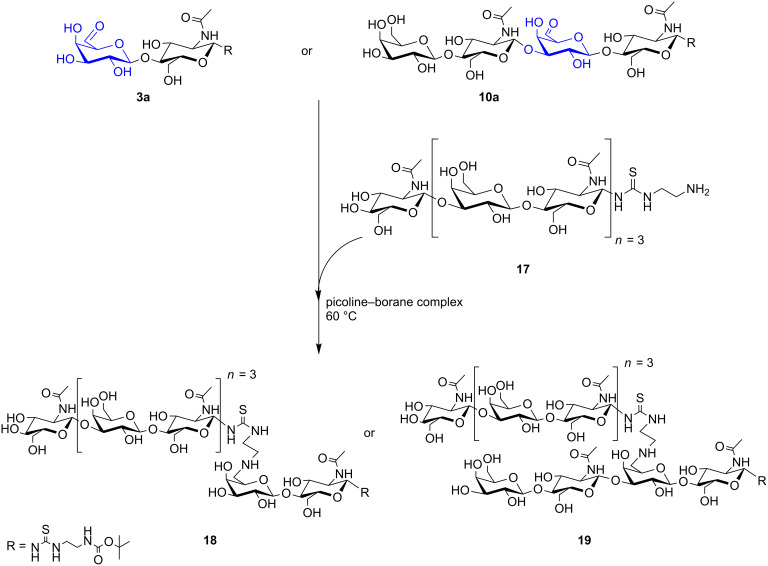
Chemical conjugation of oxidised poly-LacNAc oligomers with heptasaccharide–linker–NH_2_ (**17**) by reductive amination. Beside the main products (**18** and **19**) several byproducts are formed.

We here investigate the oxidation of LacNAc and LacDiNAc terminated poly-*N*-acetyllactosamine glycans, recently synthesised by our group [11,45], with subsequent chemical conversion of the 6-aldehyde group to the corresponding α,β-unsaturated aldehyde by base-catalysed β-elimination (Scheme 1). The galactose 6-aldehydes of poly-LacNAc oligomers were chemically converted to their corresponding α,β-unsaturated aldehydes under elevated temperature at alkaline pH in almost quantitative yield. In this way defined oxidised poly-LacNAc oligomer structures are easily obtained by galactose oxidase treatment and subsequent heating of the reaction mixture. The corresponding 6-aldehyde LacNAc oligomers were used as substrates in further elongation reactions with glycosyltransferases (Scheme 2). Remarkably, the terminally oxidised poly-LacNAc oligomers could be elongated by β3GlcNAc-transferase and subsequently by β4Gal-transferase. These results pave the way for the synthesis of a set of novel, modified poly-LacNAc glycan structures containing terminally and/or internally oxidised galactose residues for further chemical conversions. As an example, the 6-aldehydes of poly-LacNAc oligomers were converted with (+)-biotinamidohexanoic acid hydrazide (BACH), resulting in biotin-labelled poly-LacNAc glycans (Scheme 3). Moreover, the amine group obtained after deprotection of the NH_2_-linker of poly-LacNAc oligomers was utilised for the reductive amination of 6-aldehyde groups of oxidised poly-LacNAc oligomers (Scheme 4). In this way, chemically branched poly-LacNAc glycans are synthesised that resemble natural, branched poly-LacNAc glycans (I-antigens) of glycoproteins and glycolipids.

## Results and Discussion

### Evaluation of Gal- and GalNAc terminated poly-LacNAc oligomers as substrates of galactose oxidase

The activity of galactose oxidase from *Dactylium dendroides* with LacNAc **1a**, Gal-terminated poly-LacNAc oligosaccharides **1b**–**d** and LacDiNAc **2** (Scheme 1) was investigated by a photometric-activity assay and by HPLC analysis. Compared to methyl β-D-galactopyranoside the galactose oxidase showed a relative activity of about 4% for all tested saccharides (**1a**–**d** and **2** were measured at a standard concentration of 1 mM glycan, Figure S2 in Supporting Information File 1), which is sufficient for the semipreparative production of oxidised products. The methyl β-galactoside was chosen as a reference substance because of the comparable linkage at the anomeric C-atom and its known high activity [38]. No activity could be seen with odd-numbered (GlcNAc-terminated) poly-LacNAc–linker–*t-*Boc oligosaccharides, either by photometric or by HPLC analysis, even after long reaction times (data not shown). This confirms that galactose oxidase accepts only terminal galactose residues of poly-LacNAc glycans, as previously concluded for the oxidation of raffinose and plant arabinogalactan polysaccharides [36,46]. Standard enzymatic reactions were performed in sodium phosphate buffer pH 6 and analysed by HPLC after the enzymatic reaction was stopped at 95 °C at different reaction times. Three main products could be detected with all tested substrates (Scheme 1, Figure S3 in Supporting Information File 1). The products from the conversion of the tetrasaccharide **1b** and LacDiNAc **2** could be assigned according to their molecular mass as the corresponding aldehydes **3b** and **4**, the corresponding α,β-unsaturated aldehyde products **7b** and **8**, and the galacturonic acid products **5b** and **6**, as depicted in Table S1 in Supporting Information File 1 [42,44]. The formation of the corresponding uronic acids, whether chemically or enzymatically catalysed, was not further investigated. The formation of the 6-aldehydes **3a** and **3c** and the α,β-unsaturated aldehydes **7a** and **7c** for the conversion of **1a** and **1c**, was also confirmed by ESI–MS analysis (Table S1 and Figure S13 in Supporting Information File 1). Conversion of **1d** (Figure S4 in Supporting Information File 1, compared to **1c**) was followed by HPLC and gave similar product patterns to those seen for **1a**, **1b** and **1c**.

However, we asked if β-elimination is catalysed by galactose oxidase or is chemically driven by the applied reaction conditions. The products (88% **3b**, 10% **5b** and 2% **7b**) obtained after conversion of **1b** and termination of the galactose oxidase reaction by heat were incubated in 50 mM sodium phosphate buffer (varying pH as indicated) at different temperatures for different time periods, without the addition of enzyme (Figure S5 in Supporting Information File 1). We observed that the α,β-unsaturated aldehyde **7b** is formed independently of the enzyme. We concluded that dehydration of the 6-aldehyde **3b** occurs chemically at high temperatures and alkaline pH values. Similar reactions at elevated temperatures and different pH values were previously described for the synthesis of α,β-unsaturated aldehydes of α,β-galactosides, lactose, and raffinose [36,42,47]. In summary, the detailed product analysis by HPLC and ESI–MS provides evidence for the chemical conversion of the aldehyde to the corresponding α,β-unsaturated aldehyde by base-catalysed β-elimination.

### Optimisation of galactose oxidase reaction with poly-LacNAc glycans as substrates for the synthesis of 6-aldehydes

In the following experiments the oxidation of **1b** was optimised for the formation of the 6-aldehyde **3b**. No significant effect of pH (between 5 and 8) could be detected for the initial reaction rate of galactose oxidase (between 20 and 23% conversion in the first 15 min) and no marked effect on enzyme stability for the tested reaction times was detected. The pH optimum for galactose oxidase is described to be neutral to slightly acidic [48]. Different pH values were chosen for the oxidation reaction in previous studies [33,42,44,49]. We found a small preference for the formation of galacturonic acid at neutral pH, as previously described by Parikka and Tenkanen [42]. Taking these data and our results regarding the formation of acid and α,β-unsaturated aldehyde into account, we chose pH 6 for further galactose oxidase reactions.

Hydrogen peroxide is known to inhibit galactose oxidase activity [50–51]. Therefore, we tested reaction mixtures with catalase and a mixture of catalase and peroxidase, as well as peroxidase alone, for their conversion capabilities. All tested mixtures led to the same maximum, almost quantitative conversion after 24 h reaction time at pH 6, while a control sample without any additional catalase or peroxidase showed a distinctly reduced conversion. Surprisingly, the use of peroxidase alone already showed optimal conversion rates at least as good as with catalase, although a classical, appropriate donor substrate for the peroxidase was not present in the reaction. In other studies peroxidase has been shown to increase the activity of galactose oxidase, which may improve the reaction in such a way as to overcome the inhibitory effects [42,52–53]. The surface-to-volume-ratio was kept high to ensure sufficient oxygen transfer through the solvent surface. Accordingly, (semi-)preparative reactions were performed in open glass vessels to increase the transfer rate in comparison to closed ones.

Optimised reaction conditions for the conversion of **1b** yielded 94% 6-aldehyde **3b** with minimum formation of the α,β-unsaturated aldehyde **7b** (Table 1). Substrates **1a**, **1c** and **1d** gave comparable conversion rates and yields (Table 1 and Figure S4 in Supporting Information File 1). In addition, compound **2** was oxidised with basically the same conversion rate and in similar yields. Quantitative conversion of all the substrates to their corresponding 6-aldehydes without the formation of α,β-unsaturated aldehydes was achieved by avoiding heat treatment and by removal of galactose oxidase by ultrafiltration instead. In contrast, small amounts of α,β-unsaturated aldehydes were formed when the reactions were stopped with heat, as shown in Table 1.

**Table 1:** Conversion of poly-LacNAc oligomers **1a**–**d** with galactose oxidase under optimised conditions (pH 6, 24 h, 10 µmol substrate, 15.5 U galactose oxidase and 322 U peroxidase). The 6-aldehydes **3a**–**d** were obtained in 84–94% yield. A small fraction of α,β-unsaturated aldehydes **5a**–**d** was formed due to termination of the reaction by heating for 3 min at 95 °C.

substrate	peak fraction according to HPLC analysis (%)			

	educt **1a**–**d**	6-aldehyde **3a**–**d**	galacturonic acid **5a**–**d**	α,β-unsaturated aldehyde **7a**–**d**

**1a**	9	84	2	5
**1b**	0	94	1	5
**1c**	0	94	1	5
**1d**	0	92	0	8

Semipreparative synthesis (>5 mg scale) of the 6-aldehydes **3a–c** was performed for subsequent further modification. Product isolation was accomplished by preparative HPLC with overall yields (based on substrate amount) of 35 to 55%. The products **3a**, **3b** and **3c** were characterised by LC–ESI–MS (Table S1 and Figure S13 in Supporting Information File 1). **3a** and **3b** were additionally confirmed by ^1^H- and ^13^C NMR analysis (Experimental section and Table NMR 1 and Table NMR 2 in Supporting Information File 2). During evaporation of the solvents under moderate heat (40–60 °C), small amounts of α,β-unsaturated aldehyde impurities **7a**–**c** (approx. 1–5%) were formed. The corresponding LacDiNAc 6-aldehyde **4** was produced and isolated on a smaller scale (<1 mg) and analysed by LC–ESI–MS (Table S1 and Figure S13 in Supporting Information File 1).

### Chemical conversion of 6-aldehydes to their corresponding α,β-unsaturated aldehydes

**Figure 1:**
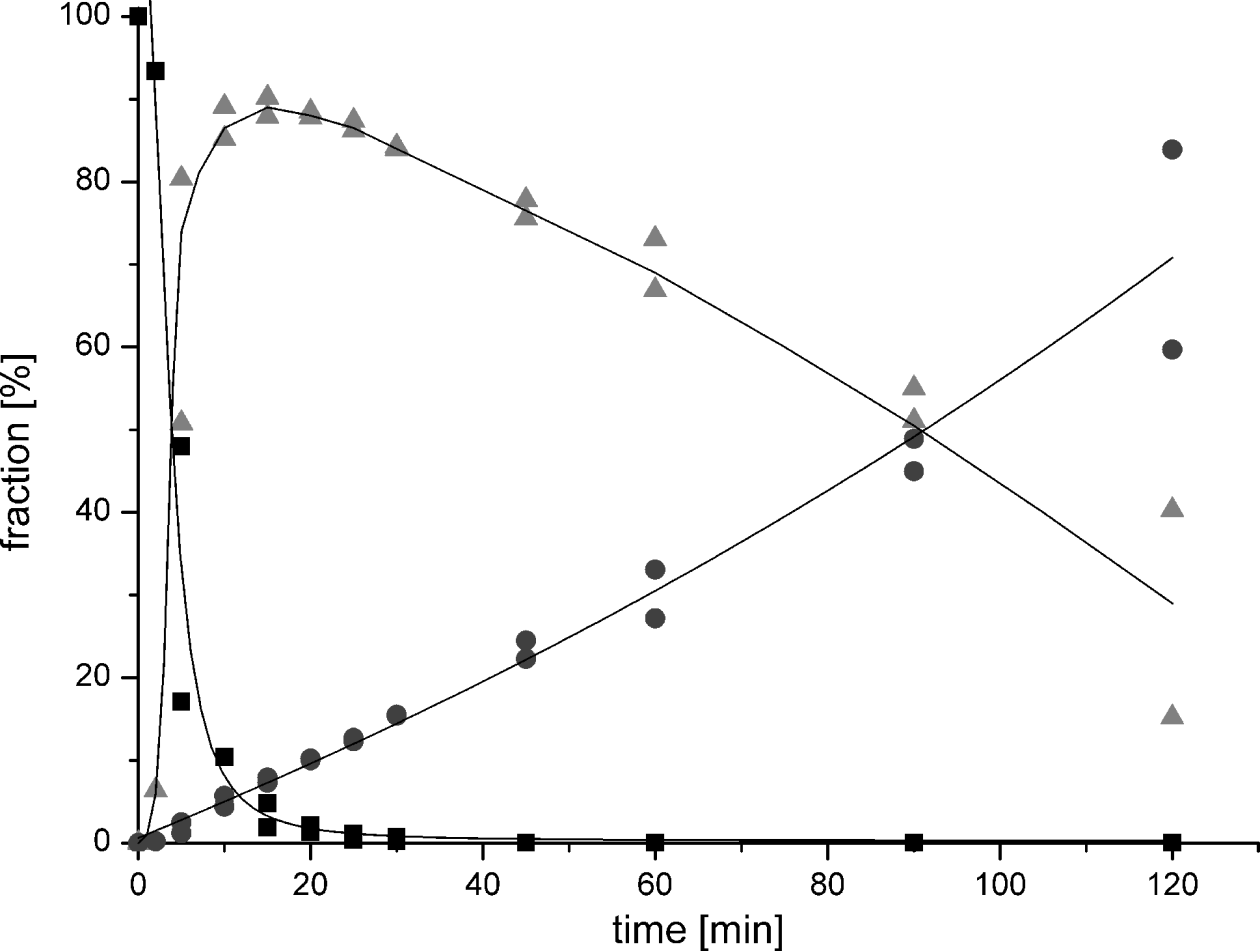
Conversion of **3b** (squares) to the corresponding α,β-unsaturated aldehyde **7b** (triangles) and side products (circles) at 95 °C and pH 9. Percentages of the products were calculated by integration of HPLC signals at 254 nm in relation to the sum of all peak integrals at 254 nm. Shown are single values of two independent conversions.

As the α,β-unsaturated aldehydes (**7a**–**d**) may also be interesting products for the evaluation of protein–glycan interactions, we decided to produce these for further analysis. To characterise and optimise the chemical conversion of 6-aldehydes to their corresponding α,β-unsaturated aldehydes (Scheme 1) the time course of the conversion of the 6-aldehydes **3a** and **3b** at pH 9 and 95 °C was monitored. HPLC analyses revealed the formation of the desired α,β-unsaturated aldehydes and several separated side products (Figure S6 in Supporting Information File 1). Complete conversion of **3a** (Figure S7 in Supporting Information File 1) and **3b** (Figure 1) was achieved within 30 min reaction time at pH 9 and 95 °C. After 15–20 min reaction time the product mixture contained up to 90% α,β-unsaturated aldehyde. However, with longer incubation the fraction of side products increased. Schoevaart et al. assumed the formation of more than 30 products under conditions of heat, with increased production rates at alkaline pH [43]. Therefore the side products were not further investigated.

The semipreparative synthesis of the α,β-unsaturated aldehydes **7a**–**c** was accomplished by the conversion of **3a**–**c** at 95 °C at pH 9 for 15 min. The products were isolated by preparative HPLC and characterised by LC–ESI–MS (Table S1 and Figure S13 in Supporting Information File 1). ^1^H- and ^13^C NMR analysis (Table NMR 3 and Table NMR 4 in Supporting Information File 2) was performed for **7a** and **7b**. The corresponding LacDiNAc α,β-unsaturated aldehyde **8** was synthesised on an analytical scale and analysed by ESI–MS (Table S1 and Figure S13 in Supporting Information File 1). This proves the formation of an α,β-unsaturated product also for the GalNAc-residue, which has, to the best of our knowledge, not been reported before.

### Enzymatic elongation of modified poly-LacNAc oligomers

**Figure 2:**
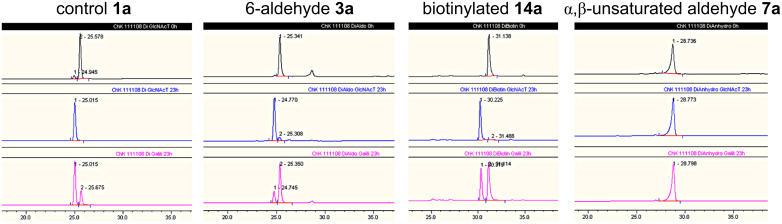
HPLC analysis for the conversion of modified LacNAc oligomers **3a, 15a**, and **7a** with β3-GlcNAc-transferase and MBP-His_6_-α3Gal-transferase. LacNAc–linker–*t-*Boc **1a** served as control. The upper panel (black) shows the substrates under the initial reaction conditions. The middle panel (blue) depicts the conversion with β3-GlcNAc-transferase after 23 h and the lower panel (magenta) the conversion with MBP-His_6_-α3Gal-transferase after 23 h. The 6-aldehyde of LacNAc (**3a**) and the C6-biotinylated LacNAc (**15a**) are substrates for enzymatic elongation, while the α,β-unsaturated aldehyde derivative of LacNAc (**7a**) is not accepted by either glycosyltransferase.

The 6-aldehydes **3a**,**b** (see Scheme 2A for **3a**), the α,β-unsaturated aldehydes **7a**,**b**, and the biotinylated poly-LacNAc glycans **15a**,**b** (see Scheme 3B) were tested as substrates for further enzymatic conversion with β3GlcNAc-transferase and MBP-His_6_-α3Gal-transferase [45,54] on an analytical scale. HPLC analysis indicated that **3a**,**b** as well as **15a**,**b** were accepted as substrates by both glycosyltransferases (Figure 2 and Figure S8 in Supporting Information File 1). In contrast, the α,β-unsaturated aldehydes **7a**,**b** are not accepted as substrates by the tested glycosyltransferases.

The reaction of **3a** with β3GlcNAc-transferase (Scheme 2A) was analysed by ESI–MS (Table S1 and Figure S13 in Supporting Information File 1) and revealed the formation of trisaccharide **9a** with an internal 6-aldehyde group. Moreover, further elongation of **9a** by β4Gal-transferase yielded the product **10a** (Scheme 2B) as analysed by ESI–MS (Table S1 and Figure S13 in Supporting Information File 1). A second oxidation round led to the production of a tetrasaccharide that shows an internal and a terminal 6-aldehyde group (**11a**, see Scheme 2C, Table S1 and Figure S13 in Supporting Information File 1). These results open a range of new possibilities to synthesise poly-LacNAc glycans with internally and/or terminally modified galactose moieties.

### Labelling of 6-aldehydes of poly-LacNAc oligomers

The 6-aldehyde products of poly-LacNAc oligomers **3a**–**c** were further reacted with BACH (**12**) to incorporate a site-specific biotin label for subsequent detection of immobilised glycans. Conversion with **12** and subsequent reduction were performed according to a standard procedure optimised in our laboratory [33]. The reactions were followed by HPLC analysis (Figure S9 and Figure S10 in Supporting Information File 1). Conversion rates of substrates **3a**–**c** were comparable, leading to 80–85% conversion after 24 h with no improvement for longer incubation times. Reduction with Na[BH_3_(CN)] was performed in a frozen state at −20 °C leading to complete reduction after 24 h (Scheme 3A). Products **15a**–**c** were purified by preparative HPLC in yields between 32 and 72% (based on initial 6-aldehyde amount) and confirmed by ESI–MS (Table S1 and Figure S13 in Supporting Information File 1). The structures of products **15a** and **15b** were characterised by ^1^H- and ^13^C NMR analysis (Experimental section and Table NMR 5–NMR 7 in Supporting Information File 2). The product **9a**, bearing an internal aldehyde group, was also modified with **12** yielding **14a**, as shown in Scheme 3A and Figure S11 in Supporting Information File 1.

### Chemical conjugation at 6-aldehydes of modified poly-LacNAc oligomers

An especially interesting coupling reaction was performed to yield chemically branched poly-LacNAc oligomers (Scheme 4). The 6-aldehyde products **3a** as well as **10a** were used for coupling of a deprotected poly-LacNAc–linker–NH_2_ glycan (**17**), which was synthesised as described previously [45,55]. On an analytical scale, a heptasaccharide–linker–NH_2_ glycoconjugate **17** was coupled to the aldehyde groups of **3a** or **10a** by reductive amination leading to the chemically branched poly-LacNAc glycan structures **18** and **19**, respectively. Products **18** and **19** were isolated by analytical HPLC and confirmed by ESI–MS (Table S1 and Figure S13 in Supporting Information File 1). Our first experiments gave only low product yields of about 10–15% before purification, due to the formation of different side products at a reaction temperature of 60 °C and long incubation time (Figure S12 in Supporting Information File 1). No further optimisation was performed but would be required for better conversion of poly-LacNAc oligosaccharide substrates, especially regarding the reaction temperature as heating of the reaction mixture leads to the formation of α,β-unsaturated aldehydes. By branching, the avidity of glycans has been shown to increase, which is important for the binding affinity of lectins [56–57]. The chemically branched poly-LacNAc glycans may, moreover, serve as analogues to naturally occurring I-antigens (β1,6-branched poly-LacNAc) [58–59]. The I-antigen structures have so far been synthesised by chemical routes [60–61]. A similar coupling of oligosaccharides has already been shown by Rodriguez et al. on peptides, but no branching site in the glycans was introduced in their study [35]. In summary, these novel neo-glycoconjugates are interesting tools for extensive binding studies of a variety of glycan binding proteins (lectins, antibodies) and for possible therapeutic applications.

## Conclusion

Novel poly-LacNAc derivatives have been produced on a micro-gram scale, which can be used for binding studies with different glycan-binding proteins, for the evaluation of binding mechanisms and biological functions. Our present study gives further insight into the reaction of galactose oxidase with poly-LacNAc substrates and has led to optimised reaction conditions, proving also the chemical conversion of 6-aldehydes under alkaline conditions to the corresponding α,β-unsaturated aldehydes. By means of ultrafiltration instead of heating, at moderate pH values, the chemical formation of α,β-unsaturated aldehydes was avoided, giving rise to a series of poly-LacNAc oligomer 6-aldehydes as a novel class of neo-glycoconjugates. We here also demonstrate, for the first time, that poly-LacNAc oligomers with terminal galactose 6-aldehyde moieties are substrates for glycosyltransferases. In this way, further poly-LacNAc glycans carrying terminal and/or internal galactose 6-aldehyde units can be built up. Incorporation of the aldehyde group gives rise to further modification with different amines, hydroxylamines, and hydrazides. Thereby modifications that introduce various chemical properties can be incorporated and tested for their influence on protein binding and biological function. The synthesised products could be valuable tools to follow glycan immobilisation on biomaterial surfaces. Preliminary results already demonstrate that biotinylated poly-LacNAc oligosaccharides (with deprotected NH_2_-linker) are detectable by streptavidin-peroxidase-detection after immobilisation on amine-reactive microtiter plates (data not shown). These products offer also the possibility of direct comparison between binding to soluble and immobilised poly-LacNAc glycans. This is especially important as it was shown that the presentation mode and the valency of glycans have a high impact on protein-binding regulation [62–63]. Binding analysis of different galectins to these novel poly-LacNAc derivatives is currently under investigation.

The production of poly-LacNAc chains with one or more modified galactose residues will allow further insight in detailed protein-glycan binding mechanisms. In the past mainly glycan-modified poly-LacNAc structures have been used to evaluate galectin affinity for internal and terminal galactose moieties [7,64]. In contrast, modified poly-LacNAc structures synthesised in this study could refine binding analysis by the incorporation of bulky residues, such as biotin, or more subtle modifications, such as an aldehyde group [65–66]. Terminal and internal aldehyde groups could be further used for the introduction of different functionalities, such as alkyne or azide groups, to facilitate orthogonal click-chemistry reactions [67]. Moreover, branched poly-LacNAc glycan structures were synthesised, which are interesting binding ligands for a variety of glycan-binding proteins. These novel chemically branched structures may serve as analogues for branched N-glycans.

## Experimental

### Materials

Galactose oxidase was purchased from Worthington (Lakewood, NJ) and peroxidase from Merck (Darmstadt, Germany). Restriction enzymes and alkaline phosphatase were supplied by Fermentas (St. Leon Roth, Germany). The used chemicals were purchased either from Sigma-Aldrich (Taufkirchen, Germany) or Carl Roth (Karlsruhe, Germany) and used without further purification.

### Analytical methods

#### Chromatography and mass spectrometry

Analytical HPLC measurements were carried out on a Dionex LC–MS System using LiChrospher® 100RP 18-5µ EC or Multokrom 100-5 C18 250 × 4 mm (both CS Chromatographie, Langerwehe, Germany) columns. The following methods were used: A: Isocratic separation of poly-LacNAc–linker–*t*-Boc glycans by using 15% MeCN in water with 0.1% formic acid added as a mobile phase at 0.5 mL/min flow rate. B: Gradient separation of LacDiNAc-compounds by applying a gradient from 11–50% MeCN in water over a time course of 50 min at a flow rate of 0.5 mL/min. C: Gradient separation of oxidation products was performed by the use of 10–15% MeCN in water at 0.5 mL/min flow rate. For preparative purification of poly-LacNAc–linker–*t*-Boc compounds, a HPLC-system from Knauer (Berlin, Germany) was used in combination with a Eurospher 100-10 C18, 250 × 20 mm column (Knauer, Berlin, Germany), which was generally described elsewhere [45]. Purification took place with the same mobile phases as described above but at a flow rate of 12.5 mL/min. The products were fractionated manually. Detection of all glycans was possible at 205 nm and 254 nm. Qualitative fast monitoring of reaction progress was also done by thin-layer chromatography on 0.2 mm silica gel 60 (ALUGRAM® Xtra SIL G/UV254, Macherey&Nagel, Germany) with either isopropanol/30% ammonia/H_2_O (7:2:1) or ethylacetate/methanol/H_2_O/acetic acid (4:2:1:0.1) as mobile phases. Isolated products were analysed by HPLC/ESI mass spectrometry (Thermo Finnigan Surveyor® MSQ™) using the following settings: negative ESI ionization, probe temperature: 400 °C; needle voltage: 4.5 kV; cone voltage: 100 V. Ten microlitres of a 0.1 mM glycan solution were concentrated on a Multospher 120 RP 18 HP-3µ (60 × 2 mm), applying a flow rate of 0.2 mL/min using 50% MeCN in water as a mobile phase. Nonpurified products **5b** and **6** were analysed from the reaction mixture by using HPLC method A without formic acid and ESI mass spectrometry as described.

#### NMR spectroscopy

NMR spectra and data may be found in Supporting Information File 2. NMR spectra were measured on a Bruker AVANCE III 600 MHz spectrometer (600.23 MHz for ^1^H, 150.93 MHz for ^13^C, and 60.82 MHz for ^15^N) in D_2_O at 30 °C. The residual signal of the solvent was used as an internal standard (δ_H_ 4.508). The carbon spectra were referenced to the signal of acetone (δ_C_ 30.50). ^1^H NMR, ^13^C NMR, COSY, HSQC, HMBC, HSQC-TOCSY, and 1-D TOCSY spectra were measured by using the standard software from the manufacturers. Chemical shifts are given in δ-scale [ppm], and coupling constants in hertz. The digital resolution enabled us to report chemical shifts of protons to three and carbon chemical shifts to two decimal places. Some hydrogen chemical shifts were read out from HSQC and are reported to two decimal places. For compounds **3a** and **3b** the anomeric configuration (β) of galactose units was determined from the *J*_H-1,H-2_ coupling constants. The glycosidic linkage was deduced by using heteronuclear correlations in the HMBC experiment (bold printed in tables) and confirmed by the downfield glycosylation shift of the involved carbons (C-4 for Glc, C-3 for Gal). Chemical shifts of GlcNAc carbons C-2 agree with N-acetylation. Because of isomerism on the NH-C=S bond, signals of H-1^A^ and CS were not detected. Chemical shifts of terminal C-6 and H-6 (87.98 and 4.861 ppm in **3a**, 88.14 and 4.955 ppm in **3b**) indicate the presence of a geminal diol (the hydrate was formed). The NMR structure assignment of **7a** and **7b** was achieved as described for the above-mentioned products **3a** and **3b**. The α,β-unsaturated aldehyde structure of compounds **7a** and **7b** was proven by the presence of a C4–C5 double bond in the terminal saccharide unit. For compounds **15a** and **15b** the HSQC-TOCSY experiment was fundamental for the assignment of particular proton spin systems of individual saccharide units, the –N(CH_2_)_2_N– and –(CH_2_)_5_N– groups of spacers, and the spin system –(CH_2_)_4_CHCH(N–)CH(N–)CH_2_– of biotin. The anomeric configuration (β) of all saccharide units was determined from the *J*_H-1,H-2_ coupling constants. Chemical shifts of GlcNAc carbons C-2 agree with *N*-acetylation. Because of isomerism on the NH–C=S bond, protons H-1^A^ resonate as two broad singlets. The above-mentioned spin systems were put together by using information extracted from the ^1^H,^13^C HMBC experiment (diagnostic correlations are bold printed in the Tables NMR 5 and NMR 6 in Supporting Information File 2). The glycosidic linkage was also demonstrated by the downfield glycosylation shift of the involved carbons (C-4 for Glc, C-3 for Gal). The structure of **15b** was supported by the ^1^H,^15^N HMBC experiment. The extracted ^1^H,^15^N contacts of seven nitrogen atoms (see the Table NMR 7 in Supporting Information File 2) confirmed the connection of particular spin systems; the remaining three nitrogens in the molecule were not detected.

### Synthesis and isolation of linear poly-LacNAc–linker–*t*-Boc glycans

Linear poly-LacNAc–linker–*t-*Boc oligomers with defined chain length (**1a**–**d** and protected **17**) were synthesised as described previously with minor variations [45]. Briefly, two Leloir-glycosyltransferases, human β1,4-galactosyltransferase-1 (β4Gal-transferase) [68] and the β1,3-*N*-acetylglucosaminyltransferase from *Helicobacter pylori* (β3GlcNAc-transferase) [69], as well as UDP-Glc/GlcNAc 4'-epimerase from *Campylobacter jejuni* [70] were recombinantly produced in *E. coli,* purified and combined in a one-pot-synthesis. LacNAc–linker–*t-*Boc (5 mM), synthesised as described previously [45,55], was incubated as starting acceptor substrate together with UDP-α-D-glucose (UDP-Glc) and UDP-α-D-*N*-acetylglucosamine (UDP-GlcNAc) as donor substrates in 100 mM HEPES-NaOH-buffer pH 7.2, 25 mM KCl, 1 mM DTT, 1 mM MnCl_2_, 1 mM MgCl_2_, 2 U/mL alkaline phosphatase approx. 5 mU/mL β4Gal-transferase, approx. 25 mU/mL β3GlcNAc-transferase and approx. 3.5 U/mL UDP-Glc/GlcNAc 4'-epimerase for 48 h to 72 h. In order to yield even- or odd-numbered poly-LacNAc glycans, the reactions were stopped by ultrafiltration (molecular weight cut off at 30 kDa) and subsequently terminated by the addition of β4Gal-transferase, UDP-Glc/GlcNAc 4'-epimerase and UDP-Glc, or β3GlcNAc-transferase and UDP-GlcNAc, as described above, and incubation for 24 h. The isolation of single, defined poly-LacNAc–linker–*t-*Boc-glycans (**1a**–**d** and protected **17**) out of the product mixture was carried out by preparative reversed-phase HPLC (method A mobile phase). After evaporation of the solvent, the products were dissolved in water and appropriate stock solutions were stored at −20 °C. Reaction progress and final products (**1a**–**d** and protected **17**) were analysed by analytical reversed-phase HPLC applying method A and assigned by comparison to defined standards [45]. Concentration was determined by integration of the peak area and calculated against a linear calibration regression of GlcNAc–linker–*t-*Boc.

### Synthesis and isolation of LacDiNAc-*t-*Boc

LacDiNAc-*t-*Boc (**2**) was synthesised as described previously with some modifications [11]. To increase enzyme stability after purification, the buffer was exchanged to storage buffer (100 mM MOPS, 25 mM KCl, pH 6.8) and 20% glycerol was added to reduce protein precipitation. To reach an improved conversion of the acceptor substrate, UDP-GlcNAc was used in 1.25-fold excess. Reaction mixtures of 5 mM GlcNAc–linker–*t-*Boc, 6.25 mM UDP-GlcNAc, recombinant mutant human galactosyltransferase 1 (His_6_-propeptide-cat-β4-galactosyltransferase Y284L) (approx. 5 mU/mL), recombinant UDP-Glc/GlcNAc 4'-epimerase from *Campylobacter jejuni* (approx. 3.5 U/mL)*,* 2 mM MnCl_2_ and 2 U/mL alkaline phosphatase were incubated for 48 h to 72 h to achieve quantitative or almost quantitative conversion. Isolation of **2** was performed as described above by preparative reversed-phase HPLC applying the mobile phase of method B. The reaction progress and final product were analysed with HPLC, method B.

### Deprotection of heptasaccharide–linker–NH_2_: synthesis of **17**

Deprotection of the poly-LacNAc oligomer (**17**) was performed as described previously, except that sugars were not lyophilised but instead dissolved in water and stored at −20 °C [55]. Deprotection was controlled by HPLC using method A. As for protected glycans, the concentration was determined by integration of the peak area and calculated against the GlcNAc–linker–*t-*Boc standard regression curve.

### Photometric galactose oxidase activity assay

Galactose oxidase activity was determined by the oxidation of 2,2'-azino-bis(3-ethylbenzothiazolin-6-sulfonic acid) (ABTS) as described previously with some modifications [71]. The assay was performed on microtiter plates by using 100 µL total volume. The standard assay contained 100 mM sodium phosphate buffer pH 6 (saturated with oxygen by flushing pure oxygen into the buffer for 10 min), 2 mM ABTS, 1 mM substrate (methyl β-D-galactoside as standard, **1a–d** and **2**) and 6 U/mL peroxidase. The reaction was started by the addition of 0.5 U/mL galactose oxidase. The reaction mixture was incubated at 30 °C and an increase of absorption at 405 nm was detected. Enzyme activity was determined from the linear reaction rate.

### Oxidation of **1a**–**d** and **2**

Standard oxidation reactions were performed in oxygen-saturated sodium phosphate buffer (50 mM, pH 6, saturated by flushing pure oxygen into the buffer for 10 min). Open glass vessels were used to allow easy oxygen transfer, and the reaction mixtures were incubated at 30 °C under gentle mixing at 60 rpm for 5.5 h. For the oxidation of 10 µmol *t-*Boc-saccharide (**1a**–**d**, **2**), 15.5 U galactose oxidase and 322 U peroxidase were used. Samples were taken to control the progress of the synthesis and the reactions were stopped for 5 min at 95 °C, unless mentioned differently. After centrifugation for 20 min the supernatant was analysed as described below. Preparative batch reactions were stopped by ultrafiltration (molecular weight cut off at 10 kDa) to reduce the formation of α,β-unsaturated aldehydes. On an analytical scale, variations of the standard procedure were assessed, for example, different pH values, enzyme amounts or reaction time. For the analysis of oxidation reactions, reversed-phase HPLC utilizing method C was performed. Qualitative fast monitoring of the reaction progress was also done by TLC. Isolation of single products (**3a**–**c**, **4**) was performed by preparative HPLC as described above with the mobile phase of method C. NMR analysis confirmed the structure of products **3a** (Figure NMR 1 and Table NMR 1 in Supporting Information File 2) and **3b** (Figure NMR 2 and Table NMR 2 in Supporting Information File 2).

### Chemical conversion of 6-aldehydes to α,β-unsaturated aldehydes

To analyse the chemical conversion of 6-aldehydes (**3a**,**b**) to the corresponding α,β-unsaturated aldehydes (**7a**,**b**), purified aldehydes or mixtures from a galactose oxidase reaction (88% aldehyde **3b**, 2% α,β-unsaturated aldehyde **7b** and 10% acid **5b**) were incubated in 50 mM sodium phosphate buffer as indicated at different pH values and various temperatures, for specific time periods. After incubation the sample was directly cooled down on ice. Formation of α,β-unsaturated aldehyde was monitored by reversed phase HPLC with method A. The conversion for Figure 1 was calculated as


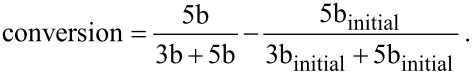


The structures of **7a** and **7b** were confirmed by NMR experiments. The corresponding spectra and data are shown in Supporting Information File 2 (Figure NMR 3 and Table NMR 3 represent **7a** and Figure NMR 4 and Table NMR 4 represent **7b**).

### Biotinylation of 6-aldehydes of poly-LacNAc oligomers

The biotinylation of 6-aldehydes **3a**–**c** was performed using biotin-amidohexanoic acid hydrazide (**12**) (BACH) according to a modified protocol from our previous work [33,49]. BACH (3.5 equiv) was added to 1 equiv isolated aldehyde (**3a**–**c**), or oxidation reaction mixture containing **3a-c**, in 25 mM sodium phosphate buffer (pH 6). The reaction was controlled directly by reversed-phase HPLC (method B) or TLC (isopropanol/30% ammonia/water 7:2:1). After 24–48 h at maximum conversion, 30 equiv of Na[BH_3_(CN)] (with reference to *t-*Boc-glycan) were added to the reaction mixture and the mixture was frozen at −20 °C. Samples were analysed again by HPLC and TLC. The product was purified by preparative HPLC as described above (mobile phase of method B), and the structure of **15a** and **15b** was confirmed by NMR analysis. The measured spectra and assigned data are found in Supporting Information File 2. Figure NMR 5 and Table NMR 5 correspond with **15a**, while Figure NMR 6 as well as Table NMR 6 and Table NMR 7 show the results for **15b**.

### Enzymatic elongation of modified poly-LacNAc oligomers

Modified di- and tetra-saccharides (**3a**,**b**, **7a**,**b** and **15a**,**b**) were incubated with β3GlcNAc-transferase and 1.2-fold excess of UDP-GlcNAc, as described above. Elongation of **3a**,**b**, **7a**,**b** and **15a**,**b** with recombinant murine α3-galactosyltransferase (MBP-His_6_-α3Gal-transferase) was performed in 100 mM MES–NaOH pH 6, 25 mM KCl, 2 mM MnCl_2_ using 1.2-fold excess of UDP-α-D-galactose (UDP-Gal). MBP-His_6_-α3Gal-transferase was cloned from an expression plasmid previously used by our group [54]. NdeI and XhoI restriction sites were incorporated by PCR. The primer sequences were: 5′-GGGAATTCCATATGGGCCATCATCATCATCATCACAGTTCGAGTGTCGAGAC-3′ and 3′-CCGTCTGTTTTCTCATATTAAACCAATCTTTATTACAGATTATTGAGCTCCGCCC-5′. The insert was digested with restriction enzymes NdeI and XhoI and ligated into the pCWori+ derivate JHP1032 described by Logan et al., which was digested with restriction enzymes NdeI and SalI, resulting in the expression vector pMisGali [69]. Expression was performed in *E.coli* BL21(DE3) in a 2.5 L Minifors fermenter (Infors HT, Bottmingen, Switzerland) by using 1.5 L TB medium containing 100 mM phosphate buffer, maintaining a pH of 7.5, and 100 µg/mL Ampicillin. After inoculation with 40 mL preparatory culture, fermentation was performed at 37 °C, 1100 rpm and 4.0 vvm mass flow. Upon reaching the stationary phase, a change of the fermentation temperature to 25 °C was followed by the addition of isopropyl β-D-thiogalactopyranoside (IPTG) yielding a concentration of 1 mM. Additionally a feed (approx. 0.1 mL/min) of 50% (v/v) glycerol in water was applied. Cultivation was terminated 2 h after IPTG addition, yielding 60 g cells (wet weight) per litre of media. Purification was done by IMAC as described elsewhere [45]. Reactions were controlled by using reversed-phase HPLC (method B). Elongation of **9a** with galactose was performed by incubation with approx. 5 mU/mL β4Gal-transferase and 1.2-fold excess of UDP-Gal, as described previously for the synthesis of poly-LacNAc glycans [45].

### Reductive amination of poly-LacNAc aldehydes **3a** and **10a** with deprotected poly-LacNAc-linker glycan **17**

Aldehyde-modified glycans (**3a** and **10a**) were incubated with two-fold excess of deprotected hepta-saccharide–linker–NH_2_ (**17**), 1.5-fold excess of picoline–borane complex and two-fold excess of acetic acid in methanol:H_2_O mixture (approx. 6:1) [72–74]. Reaction mixtures were incubated at 60 °C for 19 h. The analysis and small-scale isolation of products **18** and **19** were performed by reversed-phase HPLC using method A and ESI–MS.

## Supporting Information

File 1 Additional diagrams, HPLC chromatograms and ESI–MS spectra.

File 2 NMR data and spectra.
