# Supplementary material for: Chemo-enzymatic modification of poly-N-acetyllactosamine (LacNAc) oligomers and N,N-diacetyllactosamine (LacDiNAc) based on galactose oxidase treatment
Source: Beilstein J Org Chem. 2012 May 9;8:712–25. doi: 10.3762/bjoc.8.80 (PMC3388858; doi:10.3762/bjoc.8.80)
Supplement: File 1 — Additional diagrams, HPLC chromatograms and ESI–MS spectra. [file Beilstein_J_Org_Chem-08-712-s001.pdf]

Supporting Information

for

Chemo-enzymatic modification of poly-*N*-acetyllactosamine (LacNAc) oligomers and *N,N*-diacetyllactosamine (LacDiNAc) based on galactose oxidase treatment

Christiane E. Kupper[1], Ruben R. Rosencrantz[1], Birgit Henßen[1], Helena Pelantová[2], Stephan Thönes[1], Anna Drozdová[2], Vladimir Křen[2] and Lothar Elling*[1]

Address: [1]Laboratory for Biomaterials, Institute for Biotechnology and Helmholtz-Institute for Biomedical Engineering, RWTH Aachen University, Worringer Weg 1, Aachen, 52074, Germany and [2]Institute of Microbiology, Academy of Sciences of the Czech Republic, Videnska 1083, Prague 4, CZ 14220, Czech Republic

Email: Lothar Elling* - l.elling@biotec.rwth-aachen.de

*Corresponding author

Additional diagrams, HPLC chromatograms and ESI–MS spectra

Compounds investigated in this study

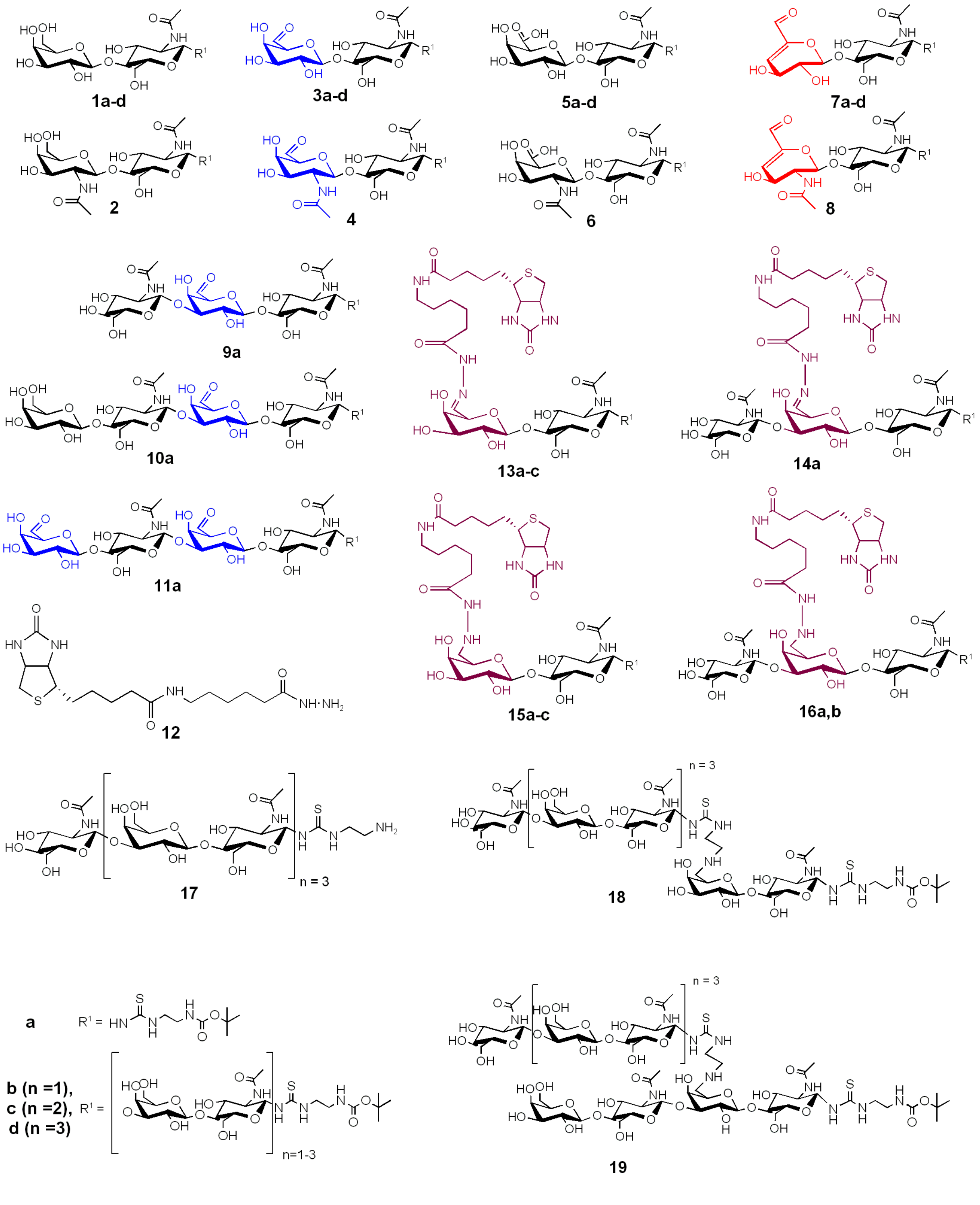

Figure S1: Mentioned compounds and their indicators throughout the text.

Table S1: Calculated and detected masses of synthesised, novel, modified poly-LacNAc oligosaccharides.

Product	Calculated mass	Calculated $[M-H]^-$	Calculated $[M-2H]^{2-}$<br>Calculated $[M-3H]^{3-}$	Detected *m/z*
3a	582.20	581.2		581.2
3b	947.35	946.35		946.3
3c	1312.5	1311.5	655.3	655.6
4	623.25	622.25		622.3
5b (not purified)	963.35	962.35		962.4
6 (not purified)	639.25	638.25		638.3
7a	564.19	563.19		563.2
7b	929.34	928.34		929.3
7c	1294.49	1293.49	646.3	646.4
8	605.24	604.24		604.2
9a	785.3	784.3		784.3
10a	947.35	946.35		946.3
11a	945.33	944.33	471.1	944.4<br>471.8
15a	937.41	936.41		936.5
15b	1302.56	1301.56	650.3	650.5
15c	1667.71	1666.71	832.9	833.2
16a[a]	1140.5	1139.5	568.8	569.4
18	1983.75	1982.75	990.4	991.5
19	2348.89	2347.89	1173<br>781.6	1174.4<br>782.3

[a]Produced by GlcNAc-elongation of **15a**, Scheme 2B.

Evaluation of Gal- and GalNAc terminated poly-LacNAc glycans as substrates of galactose oxidase

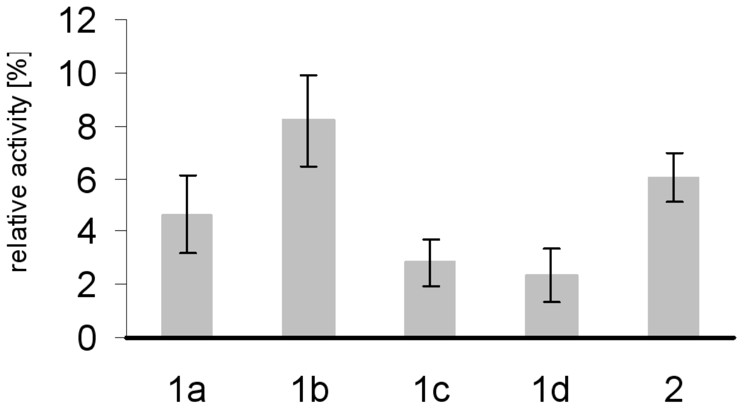

Figure S2: Relative activity of galactose oxidase in comparison to methyl-β-galactoside, measured by photometric activity assay for 1 mM of each substrate. Differences in activity measured by the photometric assay could not be confirmed by HPLC-based analysis.

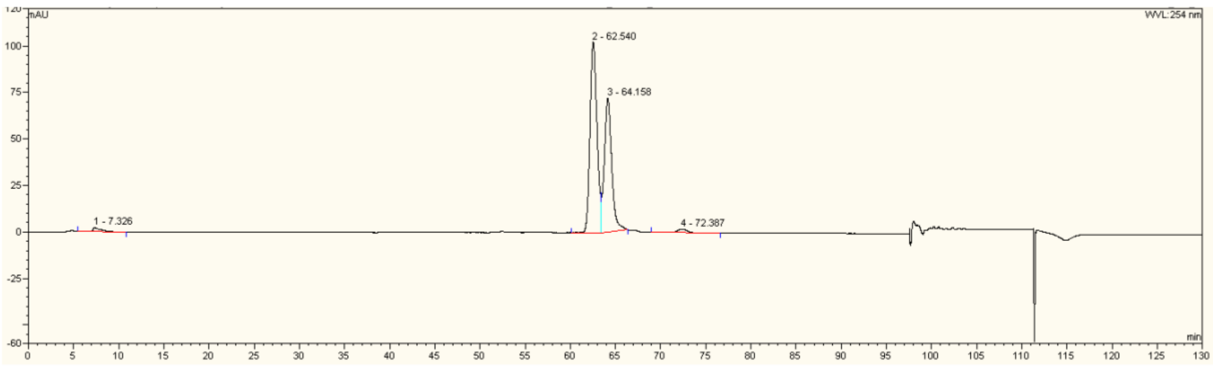

Figure S3: HPLC chromatogram of tetrasaccharide **1b** oxidation after 1 h reaction time, showing the substrate and three products, which were assigned to the corresponding products by their masses (7.3 min **5b**, 62.5 min **3b**, 64.2 min **1b** and 72.4 min **7b**).

Optimisation of galactose oxidase reaction with poly-LacNAc glycans as substrates for the synthesis of 6-aldehydes

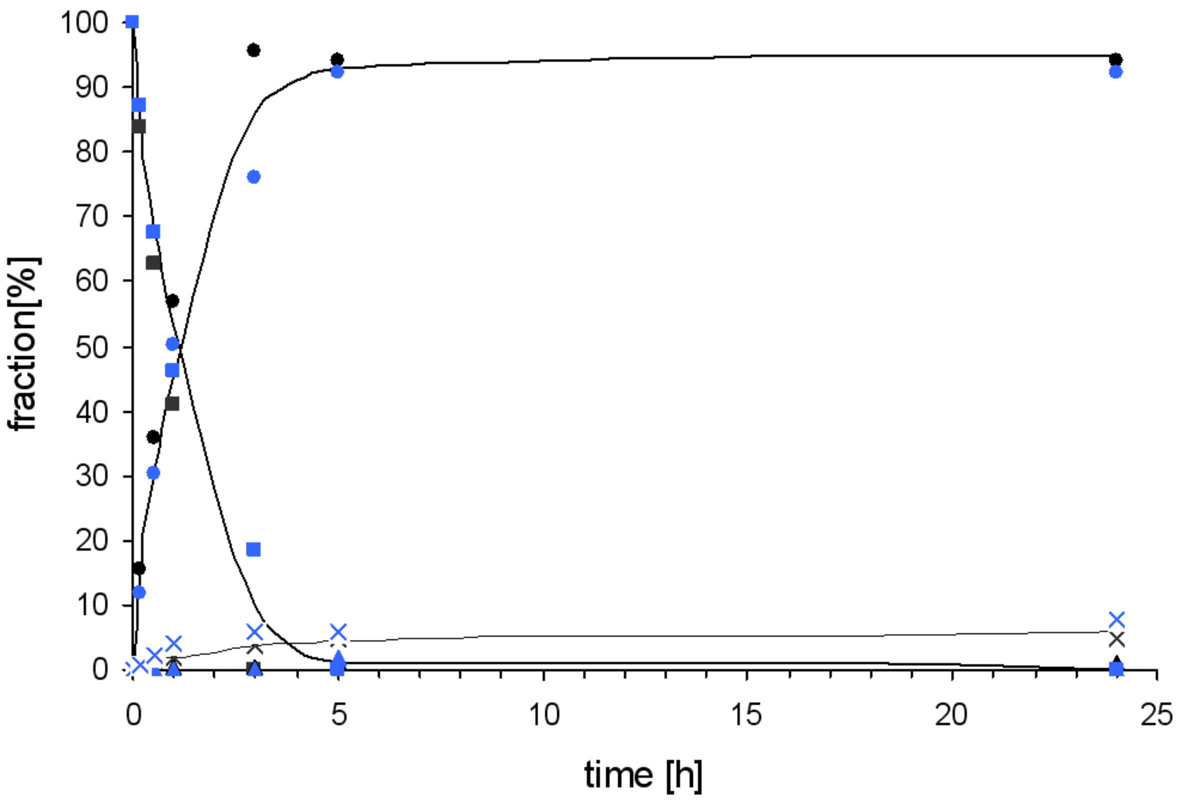

Figure S4: Conversion of **1c** and **1d** (■,■) by galactose oxidase to the corresponding aldehydes **3c,d** (●,●), galacturonic acid products (**5c,d**) (▲,▲) and α,β-unsaturated aldehydes **7c,d** (x,x) under standard conditions; reactions stopped at 95 °C for 3 min. Percentages were calculated by integration of HPLC signals at 254 nm compared to the complete integral at 254 nm.

Chemical conversion of 6-aldehydes to their corresponding α,β-unsaturated aldehydes

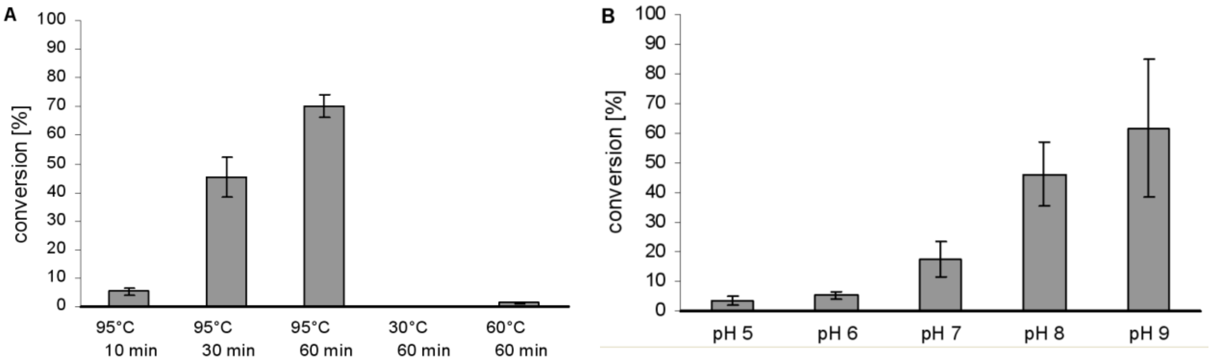

Figure S5: Conversion of the 6-aldehyde **3b** to the α,β unsaturated aldehyde **7b** (Scheme 1) in dependence of temperature, incubation time, and pH value, as analysed by HPLC.
A: All samples were incubated at pH 6 for the given time periods and temperature. Conversion of **3b** to **7b** occurs only at high temperatures.
B: Incubations were performed for 10 min at 95 °C at varying pH; our results prove the catalytic effect of basic conditions.

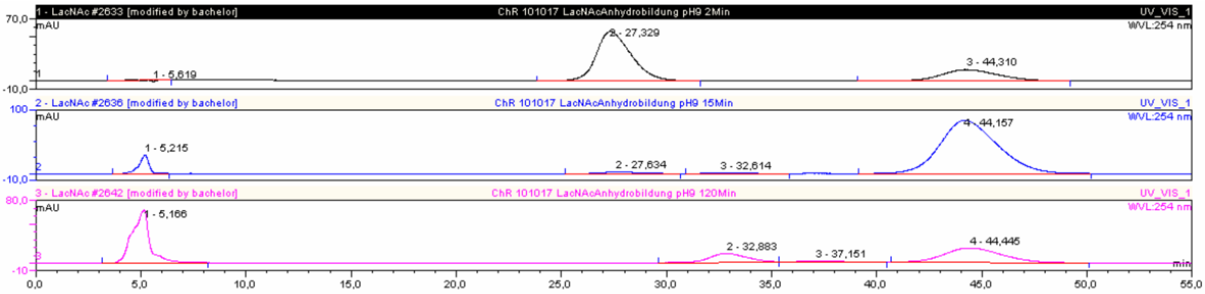

Figure S6: HPLC-chromatograms of conversion of **3a** to **7a** by heating at 95 °C at pH 9. Chromatograms are shown for 2 min, 15 min and 120 min incubation. Conversion of **3a** (27.4 min) to **7a** (44.2 min) and several side products (around 5 min, 32.8 min and 37.2 min) is shown.

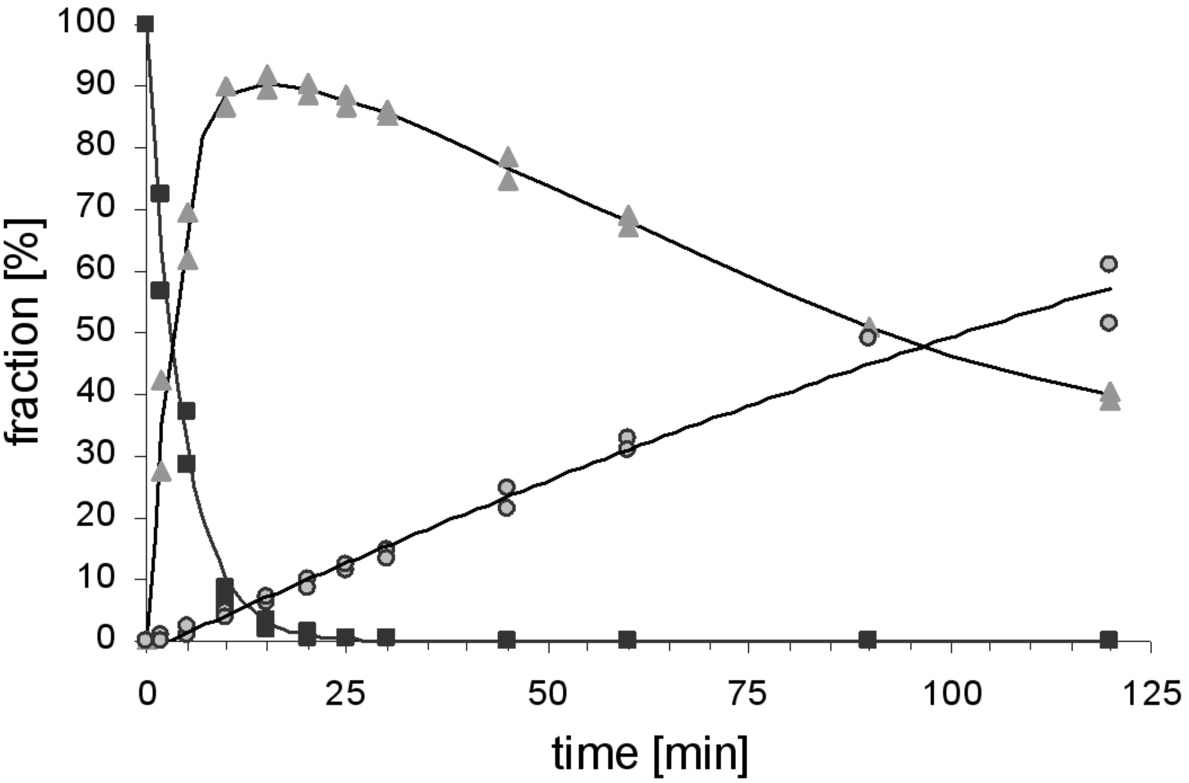

Figure S7: Conversion of **3a** (■) to the corresponding α,β-unsaturated aldehyde **7a** (▲) and side products (●) at 95 °C and pH 9. Percentages of the products were calculated by integration of HPLC signals at 254 nm in relation to the sum of all peak integrals at 254 nm. Shown are single values of two independent conversions.

Enzymatic elongation of modified poly-LacNAc glycans

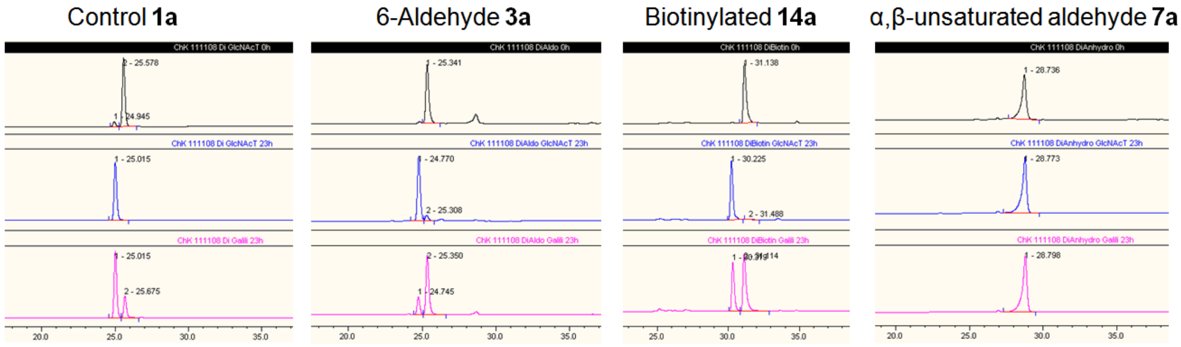

Figure S8: HPLC-chromatograms for enzymatic elongation of modified tetrasaccharide glycans **3b, 15b** and **7b** with β3-GlcNAc-transferase and α3-Gal-transferase (Galili-enzyme). Tetrasaccharide **1b** served as control. The upper (black) panel shows the substrates under the initial reaction conditions, the middle (blue) panel depicts the conversion with β3-GlcNAc-transferase after 21 h, and the bottom (magenta) panel shows the conversion with Galili enzyme after 21 h. The 6-aldehyde of the tetrasaccharide **3b** and the C6-biotinaylated tetrasaccharide **15b** are substrates for enzymatic elongation, while the α,β-unsaturated aldehyde derivative of tetrasaccharide **7b** is not a substrate for either of the glycosyltransferases. The data are not directly comparable as the enzyme activity varied slightly between different purification batches used for these two experiments.

Labelling of 6-aldehydes of poly-LacNAc glycans

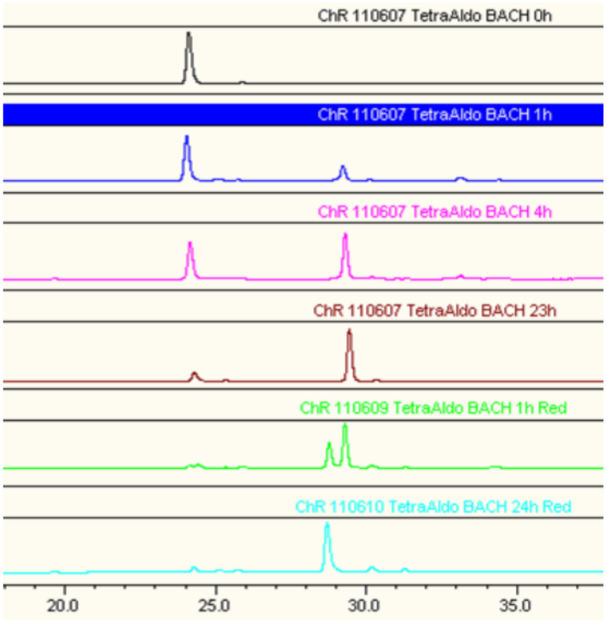

Figure S9: HPLC analysis of **3b** conversion with BACH **12** (**Scheme 3A**). Representative chromatograms of conversion and reduction are depicted: Line 1 (upper) to line 4 show reaction mixtures of **3b** (retention time approximately 24 min) with **12** (no signal at 254 nm) after 0, 1, 4 and 23 h, respectively. Biotin–hydrazone-product (**13b**) elutes after approximately 29.5 min. Line 5 and 6 show the reduction of **13b** to biotin–hydrazine (**15b**) after 1 and 24 h, respectively. A retention-time shift of approximately one minute is obvious.

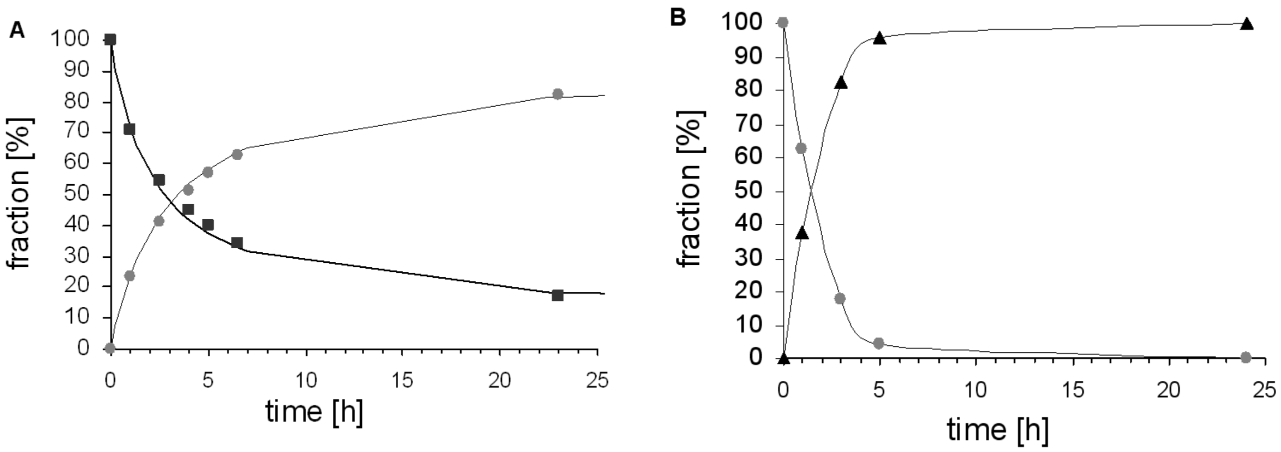

Figure S10: Time course of syntheses of **13b** and **15b** (Scheme 3A). **A:** Conversion of **3b** (■) to **13b** (●) at pH 6. Percentages were calculated by integration of HPLC signals at 254 nm compared to the complete integral at 254 nm. The conversions of 6-aldehydes **3a** and **3c** were comparable. **B:** Reduction of **13b** to **15b** by Na[BH_3(CN)] (30 equiv) at −20 °C. Percentages were calculated by integration of HPLC signals at 254 nm compared to the sum of integrals of **13b** and **15b** at 254 nm.

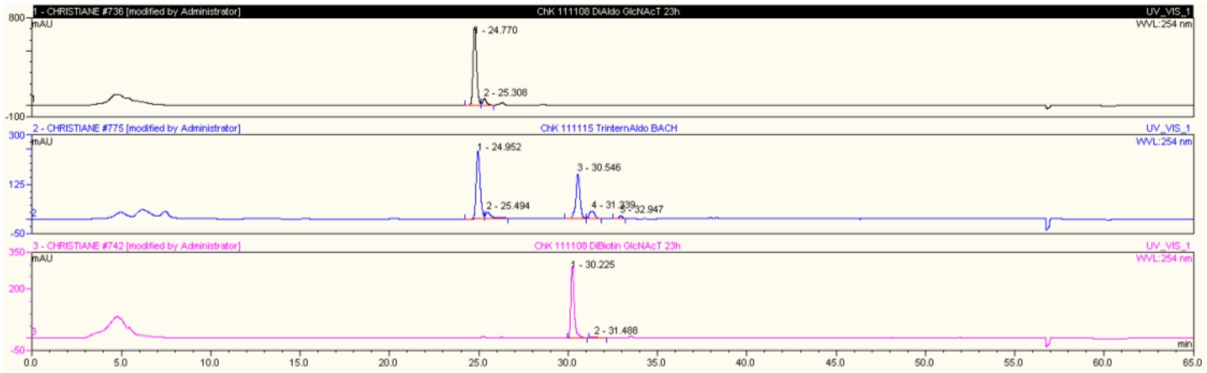

Figure S11: HPLC chromatogram of trisaccharide-6-aldehyde **9a** and its conversion with BACH **12**. In the upper panel trisaccharide-6-aldehyde **9a** (unpurified reaction mixture) is shown. **9a** (24.9 min) was incubated with **12** (not visible at 254 nm) leading to the production of **14a** (30.5 min) as shown in the middle panel. The lower panel shows the production of **16a** by enzymatic elongation of biotinylated LacNAc–linker–*t*-Boc **13a** with β3GlcNAc-transferase. The very small difference in retention time between the products in the middle and lower panel (30.5 compared to 30.2 min retention time) can be explained by the fact that the product in the middle panel (**14a**) has a hydrazone linkage, while the product in the lower panel (**16a**) has a hydrazine bond (reduced hydrazone bond).

Chemical elongation of 6-aldehydes of poly-LacNAc glycans

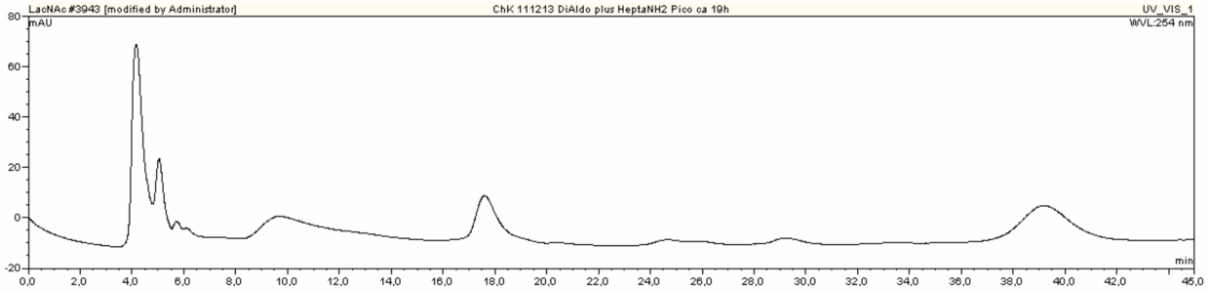

Figure S12: HPLC chromatogram of reaction of **3a** with **17** to **18** after 19 h reaction time at 60 °C. Beside the deprotected poly-LacNAc glycan (**17**, retention time approximately 4 min) and the product (**18**, retention time approximately 17.5 min), several side products can be detected. The product at a retention time of approximately 39 min can be assigned to the corresponding α,β-unsaturated aldehyde (**7a**), which could be detected in a control HPLC-chromatogram at almost the same retention time. Other side products could not be identified.

3a

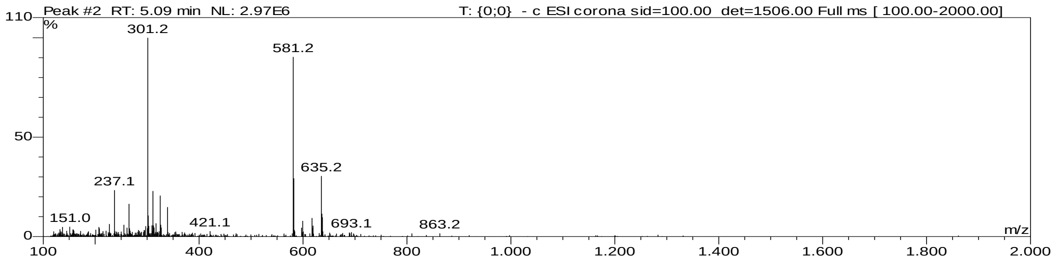
Peak #2 RT: 5.09 min NL: 2.97E6
T: {0;0} - c ESI corona sid=100.00 det=1506.00 Full ms [100.00-2000.00]
%
301.2
581.2
237.1
635.2
151.0
421.1
693.1
863.2
m/z

3b

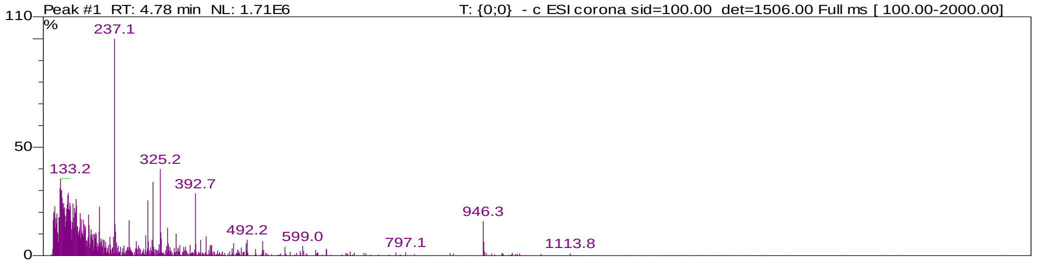
Peak #1 RT: 4.78 min NL: 1.71E6
T: {0;0} - c ESI corona sid=100.00 det=1506.00 Full ms [100.00-2000.00]
%
237.1
133.2
325.2
392.7
946.3
492.2
599.0
797.1
1113.8

3c

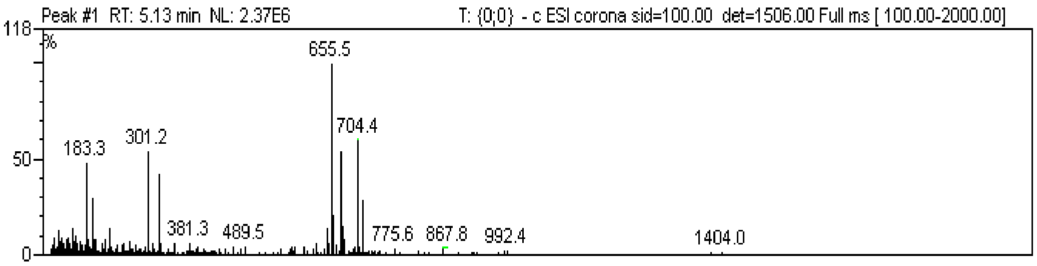
Peak #1 RT: 5.13 min NL: 2.37E6
T: {0;0} - c ESI corona sid=100.00 det=1506.00 Full ms [100.00-2000.00]
%
655.5
704.4
183.3
301.2
381.3
489.5
775.6
867.8
992.4
1404.0

4

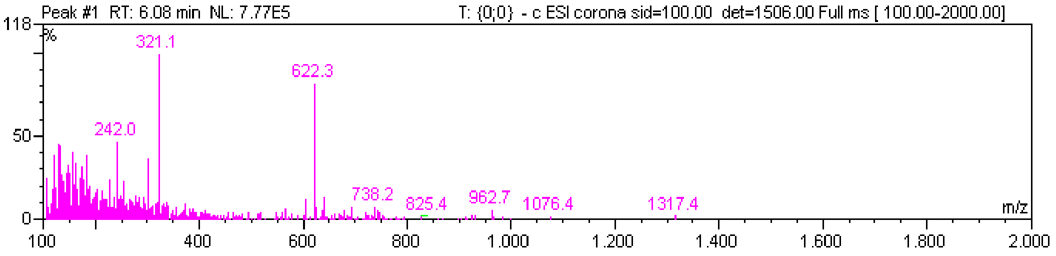
Peak #1 RT: 6.08 min NL: 7.77E5
T: {0;0} - c ESI corona sid=100.00 det=1506.00 Full ms [100.00-2000.00]
%
321.1
622.3
242.0
738.2
825.4
962.7
1076.4
1317.4
m/z

5b

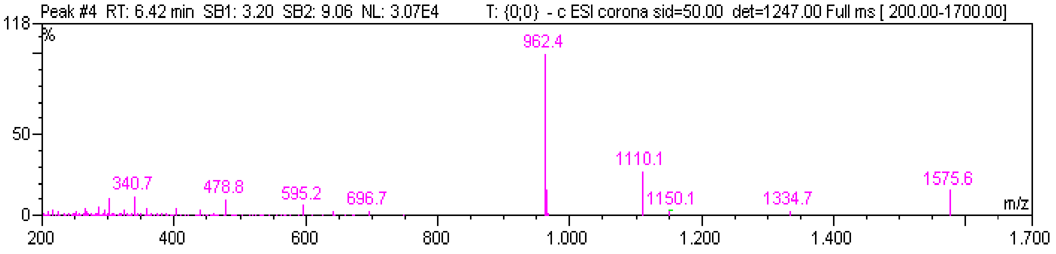
Peak #4 RT: 6.42 min SB1: 3.20 SB2: 9.06 NL: 3.07E4
T: {0;0} - c ESI corona sid=50.00 det=1247.00 Full ms [200.00-1700.00]
%
962.4
1110.1
340.7
478.8
595.2
696.7
1150.1
1334.7
1575.6
m/z

6

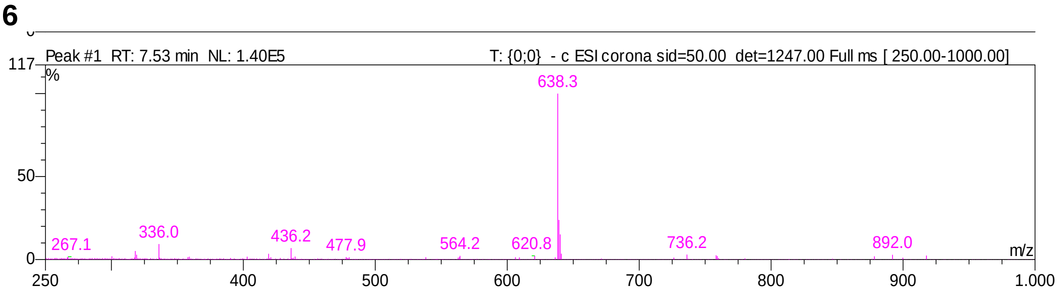
Peak #1 RT: 7.53 min NL: 1.40E5
T: {0;0} - c ESI corona sid=50.00 det=1247.00 Full ms [250.00-1000.00]
%
638.3
267.1
336.0
436.2
477.9
564.2
620.8
736.2
892.0
m/z

7a

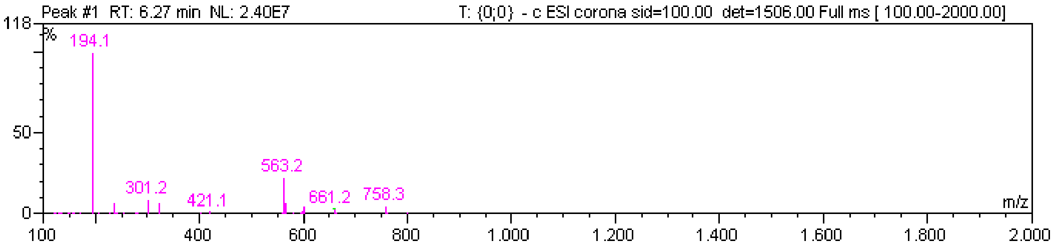

7b

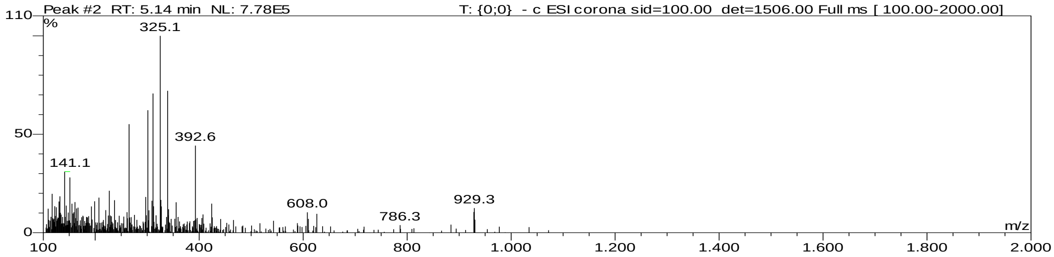

7c

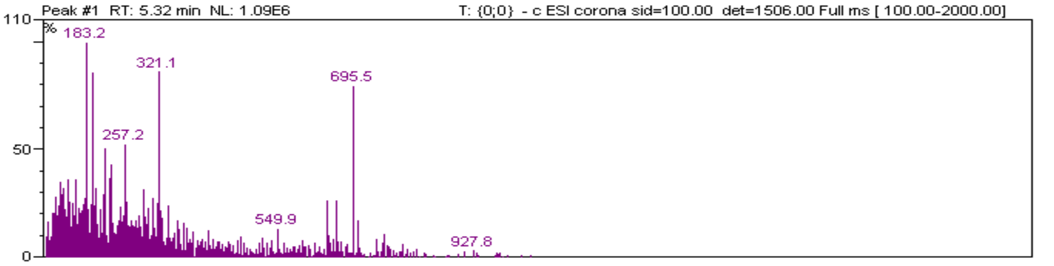

8

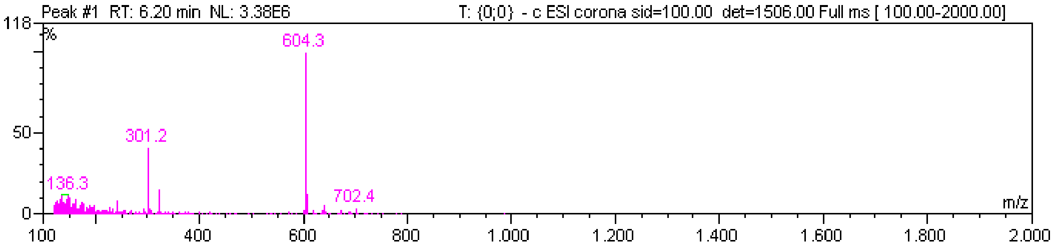

9a

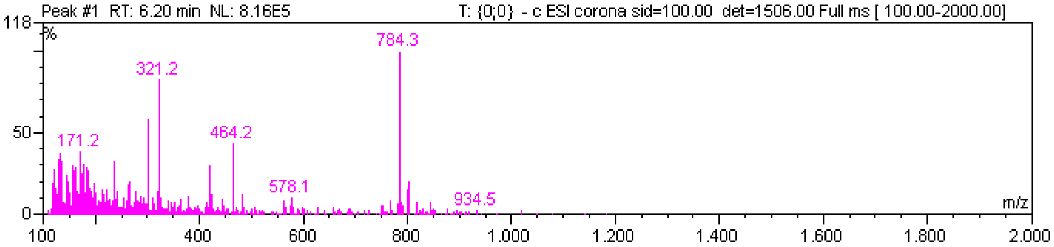

10a

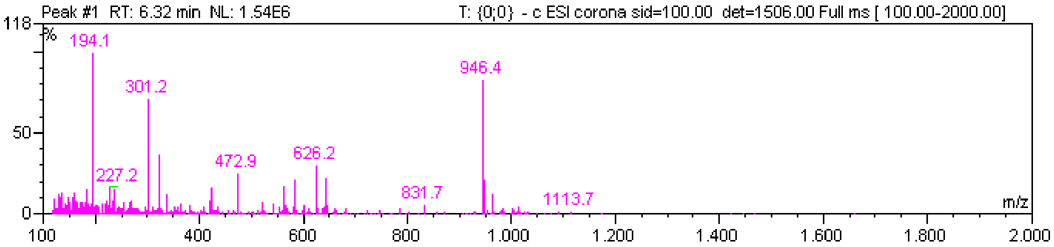

11a

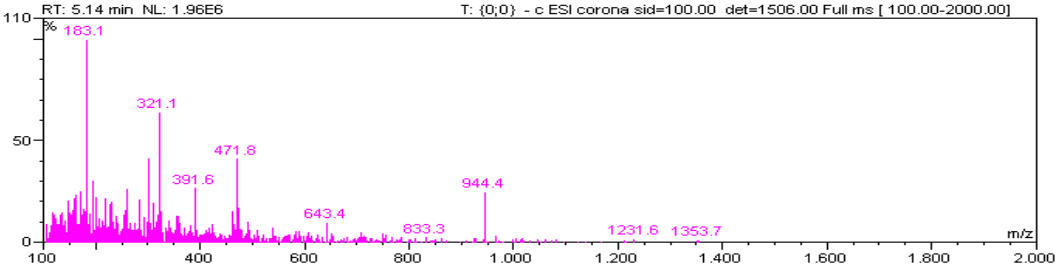

15a

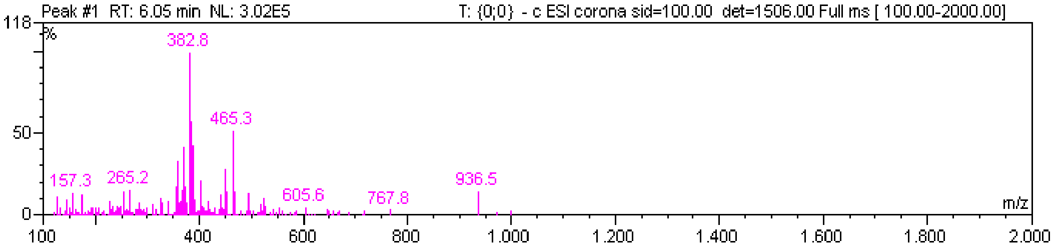

15b

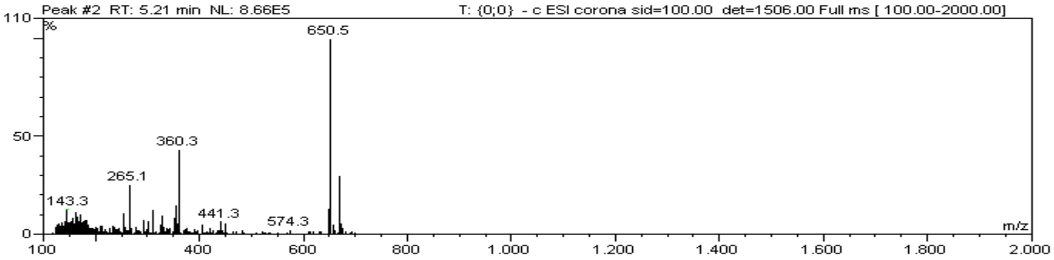

15c

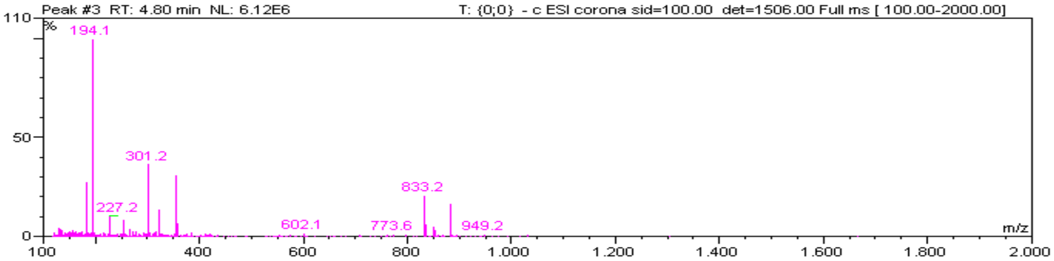

16a

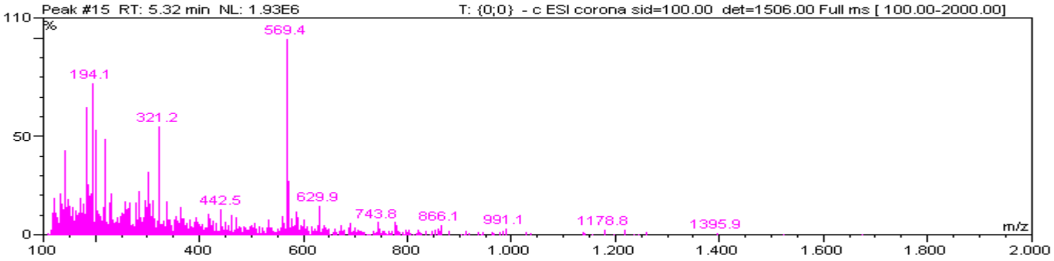

18

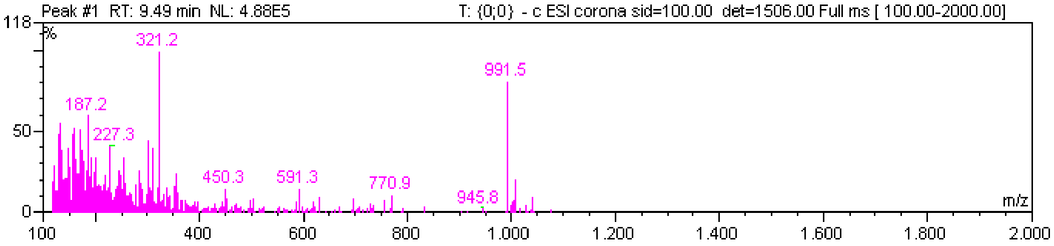

19

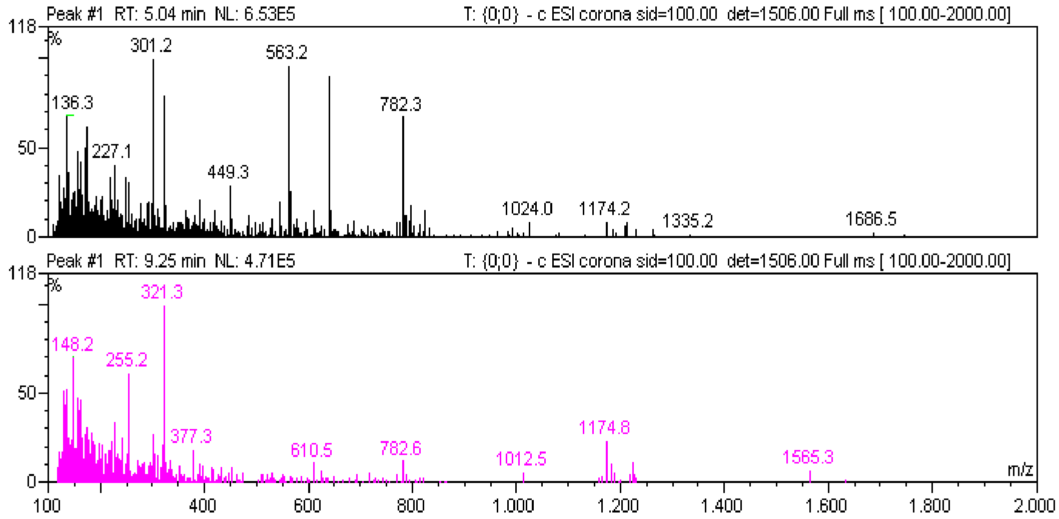

Figure S13: ESI–MS data for the different products as indicated.
