# Supplementary material for: Chemo-enzymatic modification of poly-N-acetyllactosamine (LacNAc) oligomers and N,N-diacetyllactosamine (LacDiNAc) based on galactose oxidase treatment
Source: Beilstein J Org Chem. 2012 May 9;8:712–25. doi: 10.3762/bjoc.8.80 (PMC3388858; doi:10.3762/bjoc.8.80)
Supplement: File 2 — NMR data and spectra. [file Beilstein_J_Org_Chem-08-712-s002.pdf]

Supporting Information

for

Chemo-enzymatic modification of poly-*N*-acetyllactosamine (LacNAc) oligomers and *N,N*-diacetyllactosamine (LacDiNAc) based on galactose oxidase treatment

Christiane E. Kupper[1], Ruben R. Rosencrantz[1], Birgit Henßen[1], Helena Pelantová[2], Stephan Thönes[1], Anna Drozdová[2], Vladimir Křen[2] and Lothar Elling*[1]

Address: [1]Laboratory for Biomaterials, Institute for Biotechnology and Helmholtz-Institute for Biomedical Engineering, RWTH Aachen University, Worringer Weg 1, Aachen, 52074, Germany and [2]Institute of Microbiology, Academy of Sciences of the Czech Republic, Videnska 1083, Prague 4, CZ 14220, Czech Republic

Email: Lothar Elling* - l.elling@biotec.rwth-aachen.de

*Corresponding author

NMR data and spectra

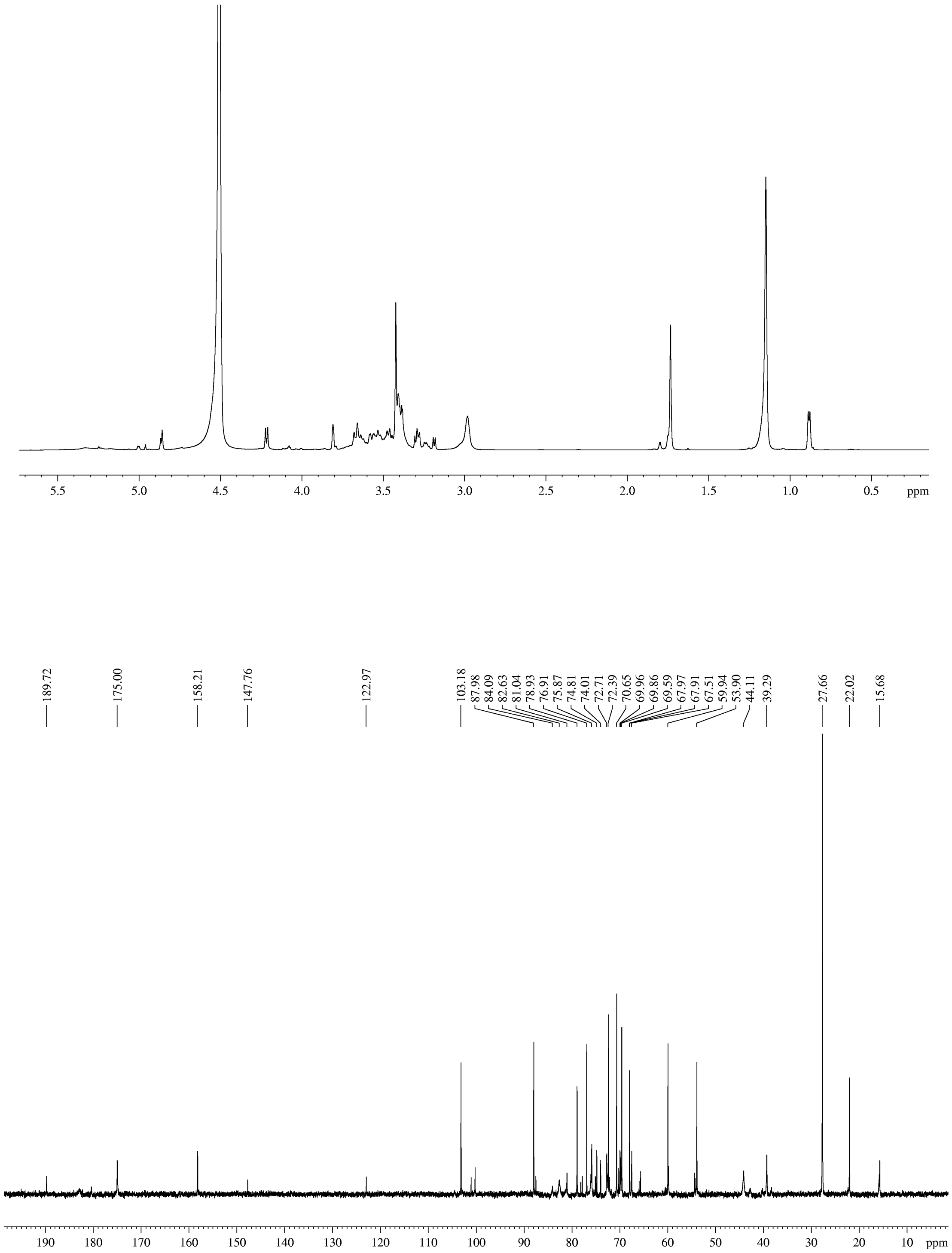

Figure NMR 1: ^{1}H and ^{13}C NMR spectra of **3a** (600.23 MHz for ^{1}H, 150.94 MHz for ^{13}C, D_2O, 30 °C).

Table NMR 1: ^{1}H and ^{13}C NMR data of **3a** (600.23 MHz for ^{1}H, 150.94 MHz for ^{13}C, D_2O, 30 °C).[a]

	assignment	δ_C	m.	δ_H	n_H	m.	*J*[Hz]	HMBC (C to H)
**Boc**	**CO**	158.21	S	-	0			2‘
	**C**	81.04	S	-	0			$(CH_3)_3$
	**$(CH_3)_3$**	27.66	Q	1.147	9	s		
**spacer**	**1‘**	44.11	T	3.389	2	m		2 w
	**2‘**	39.29	T	2.980	2	m		
	**CS**	n.d.	S	-	0			
**Glc^{A}**	**1**	82.63	D	n.d.	1			
	**2**	53.90	D	3.637	1	m		3, 4 w, 2-Ac
	**3**	72.71	D	3.532	1	dd	ΣJ = 18.0	4 w
	**4**	78.93	D	3.460	1	dd	ΣJ = 18.1	**1^{B}**, 6u, 6d
	**5**	75.87	D	3.407	1	m		4
	**6**	59.94	T	3.668	1	dd		4
				3.570	1	dd	12.5, 4.0	
	**2-Ac**	22.02	Q	1.734	3	s		
	**CO**	175.00	S	-	0			2-Ac
**Gal^{B}**	**1**	103.18	D	4.216	1	d	7.8	2, 3, 5, **4^{A}**
	**2**	70.65	D	3.291	1	dd	9.9, 7.8	1 w, 3, 4
	**3**	72.39	D	3.393	1	dm	9.9	1 w, 3, 4, 5 w
	**4**	67.97	D	3.808	1	m	ΣJ = 5.3	3, 5, 6 w
	**5**	76.91	D	3.185	1	m	ΣJ = 10.2	6
	**6**	87.98	D	4.861	1	dm	7.4, ΣJ = 1.8	4, 5

[a]w: weak signal; n.d.: not detected; u: up-field; d: down-field

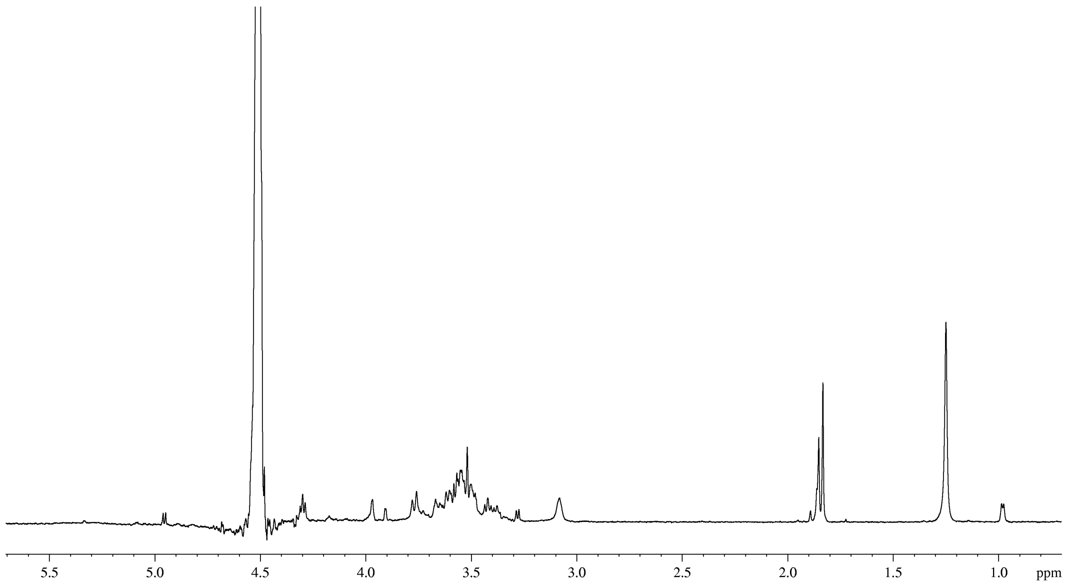

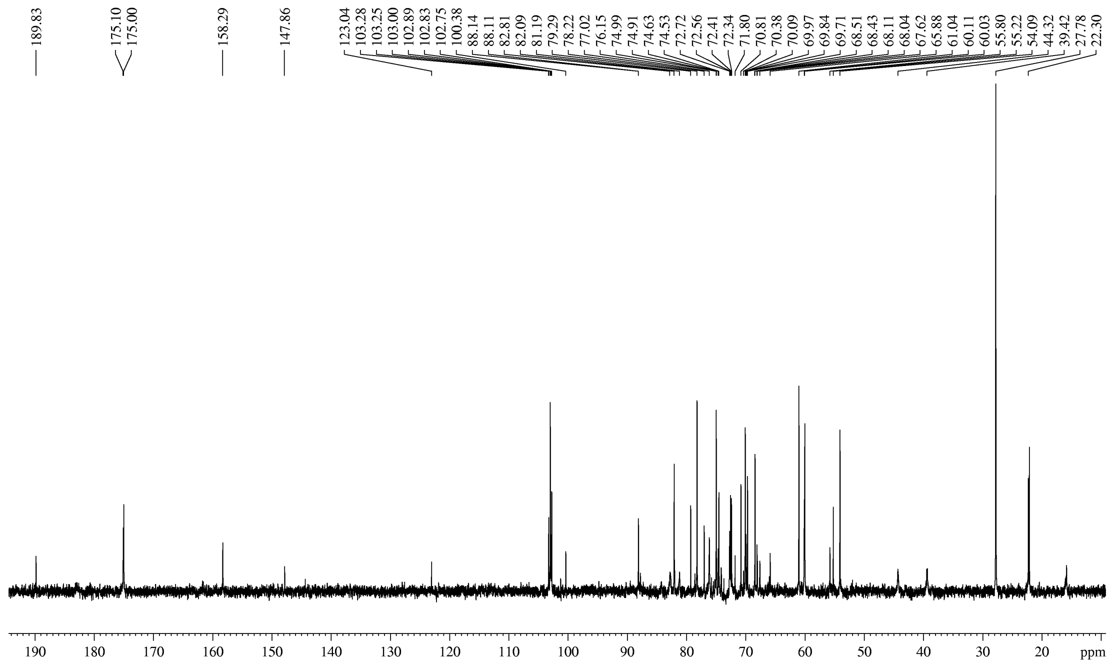

Figure NMR 2: ^{1}H and ^{13}C NMR spectra of **3b** (600.23 MHz for ^{1}H, 150.94 MHz for ^{13}C, D_2O, 30 °C).

Table NMR 2: ^{1}H and ^{13}C NMR data of **3b** (600.23 MHz for ^{1}H, 150.94 MHz for ^{13}C, D_2O, 30 °C).[a]

	assignment	δ_C	m.	δ_H	n_H	m.	*J*[Hz]	HMBC (C to H)
**Boc**	**CO**	158.29	S	-	0			
	**C**	81.19	S	-	0			$(CH_3)_3$
	**$(CH_3)_3$**	27.78	Q	1.249	9	s		
**spacer**	**1‘**	44.32	T	3.480	2	m		
	**2‘**	39.42	T	3.082	2	m		
	**CS**	n.d.	S	-	0			
**Glc^A^**	**1**	82.81	D	n.d.	-			
	**2**	54.09	D	3.73[b]	1	m		3, 4, 2-Ac
	**3**	72.72	D	3.64[b]	1	m		
	**4**	78.22	D	3.55[b]	1	m		6u, 6d, **1^B^**
	**5**	76.15	D	3.49[b]	1	m		4, 6d
	**6**	60.11	T	3.77[b]	1	m		4
				3.66[b]	1	m		
	**2-Ac**	22.13	Q	1.833	3	s		
	**CO**	175.10	S	-	0			2
**Gal^B^**	**1**	103.00	D	4.294	1	d	7.2	2, **4^A^**
	**2**	70.09	D	3.423	1	m		
	**3**	82.09	D	3.546	1	m		1, 2, 4, **1^C^**
	**4**	68.43	D	3.970	1	dm	2.4	
	**5**	74.91	D	3.54[b]	1	m		1, 4, 6
	**6**	61.04	T	3.57[b]	2	m		4, 5
**Glc^C^**	**1**	102.75	D	4.52[b]	1	m		2, **3^B^**
	**2**	55.22	D	3.62[b]	1	m		3, 2-Ac
	**3**	72.41	D	3.57[b]	1	m		
	**4**	79.29	D	3.54[b]	1	m		5, 6u, 6d, **1^D^**
	**5**	74.53	D	3.41[b]	1	m		6u, 6d
	**6**	60.03	T	3.77[b]	1	m		4
				3.66[b]	1	m		
	**2-Ac**	22.30	Q	1.852	3	s		
	**CO**	175.01	S	-	0			2, 2-Ac
**Gal^D^**	**1**	103.28	D	4.300	1	d	7.0	2, 5
	**2**	70.81	D	3.379	1	m		
	**3**	72.56	D	3.488	1	m		
	**4**	68.11	D	3.907	1	dm	2.6	6
	**5**	77.02	D	3.280	1	dm	7.4	
	**6**	88.14	D	4.955	1	d	7.4	1, 5

[a]n.d.: not detected; u: up-field; d: down-field; [b]HSQC readout

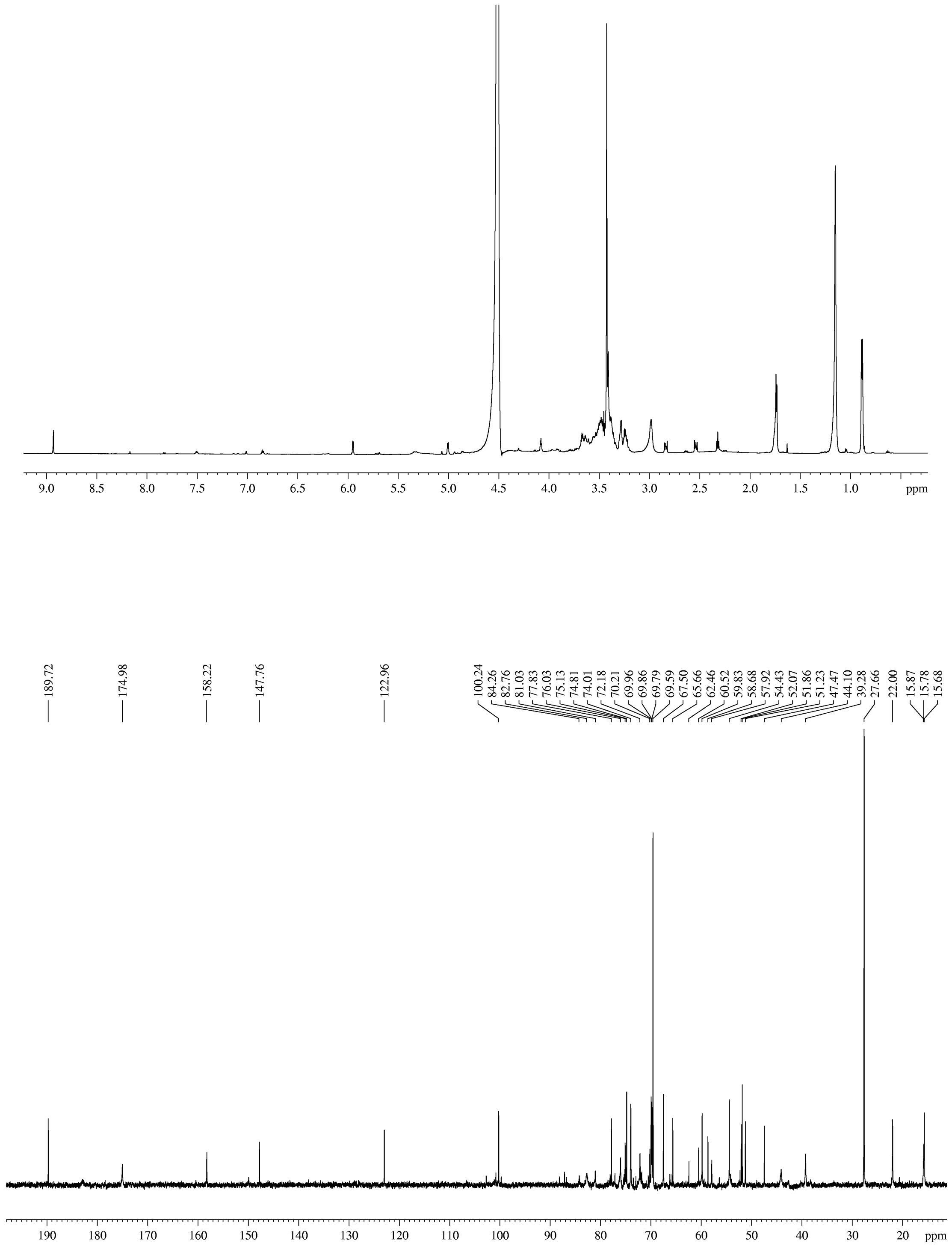

Figure NMR 3: ^{1}H and ^{13}C NMR spectra of **7a** (600.23 MHz for ^{1}H, 150.94 MHz for ^{13}C, D_2O, 30 °C).

Table NMR 3: ^{1}H and ^{13}C NMR data of **7a** (600.23 MHz for ^{1}H, 150.94 MHz for ^{13}C, D_2O, 30 °C).[a]

	assignment	δ_C	m.	δ_H	n_H	m.	*J*[Hz]	HMBC (C to H)
**Boc**	**CO**	158.22	S	-	0			2‘
	**C**	81.03	S	-	0			$(CH_3)_3$
	**$(CH_3)_3$**	27.66	Q	1.149	9	s		
**spacer**	**1‘**	44.10	T	3.39[b]	2	m		
	**2‘**	39.28	T	2.983	2	m		
	**CS**	n.d.	S	-	0			
**Glc^A**	**1**	82.76	D	n.d.	1			
	**2**	54.48	D	3.62[b]	1	m		2-Ac w
	**3**	75.13	D	3.50[b]	1	m		
	**4**	77.83	D	3.53[b]	1	m		**1^B**, 6d, 5w
	**5**	76.03	D	3.38[b]	1	m		
	**6**	59.83	T	3.65[b]	1	m		
				3.56[b]	1	m		
	**2-Ac**	22.00	Q	1.730	3	s		
	**CO**	174.98	S	-	0			2-Ac
**Gal^B**	**1**	100.24	D	5.005	1	d	5.0	2, 3, 6, **4^A**
	**2**	69.79	D	3.671	1	dd	5.0, 4.6	
	**3**	65.66	D	4.079	1	dd	4.6, 3.9	
	**4**	122.96	D	5.949	1	d	3.9	2, 3
	**5**	147.76	S	-	0			1, 3, 4, 6
	**6**	189.72	D	8.931	1	s		4, 3

[a]n.d.: not detected; u: up-field; d: down-field; [b]HSQC readout

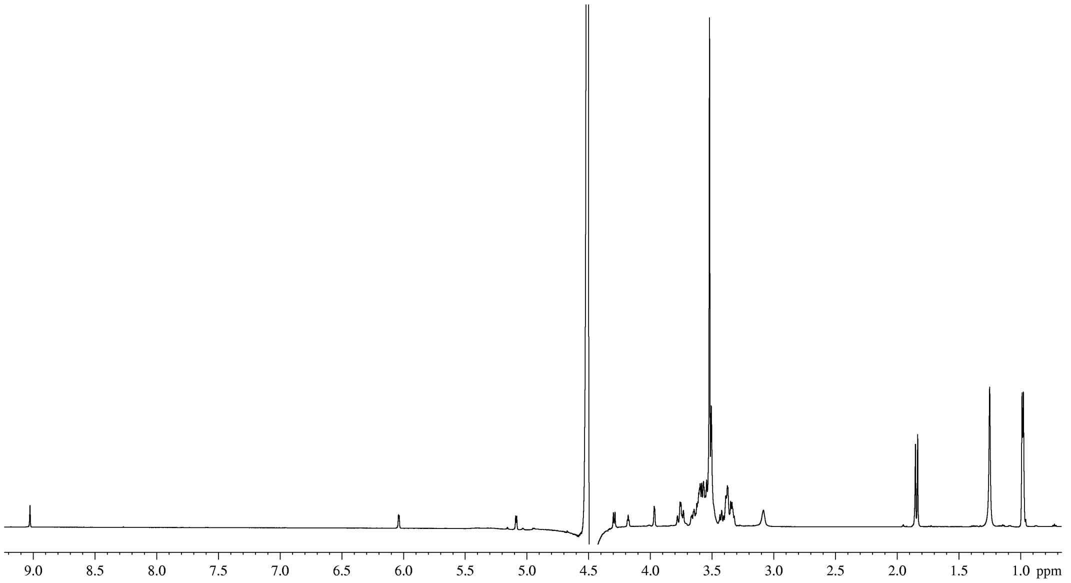

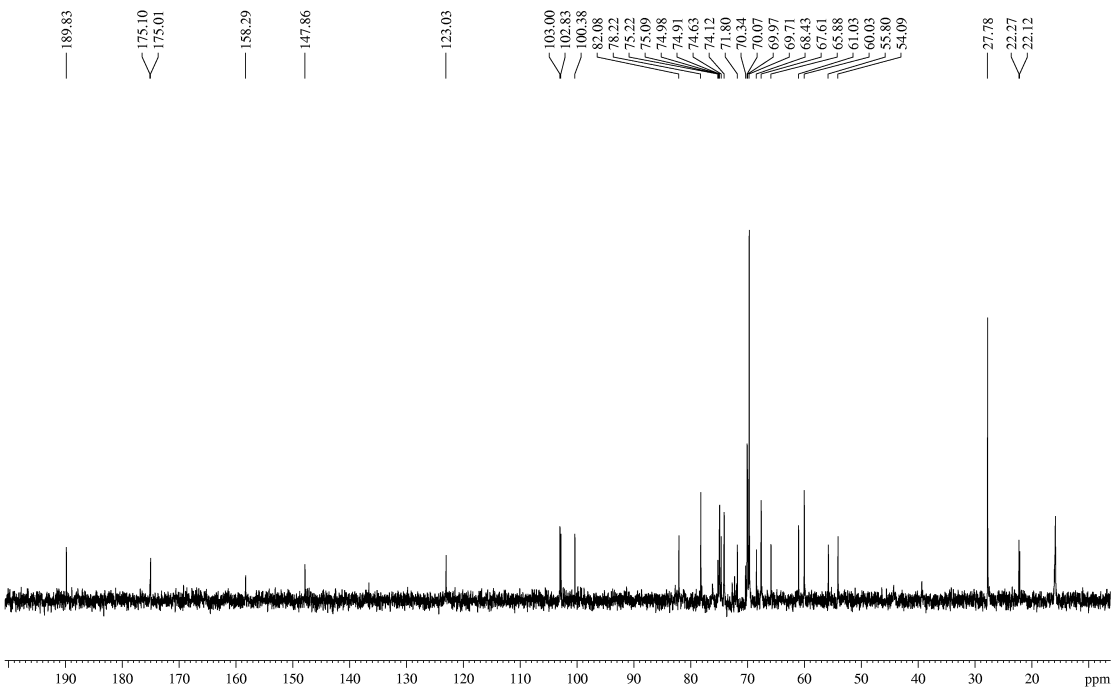

Figure NMR 4: ^{1}H and ^{13}C NMR spectra of **7b** (600.23 MHz for ^{1}H, 150.94 MHz for ^{13}C, D_2O, 30 °C).

Table NMR 4: ^{1}H and ^{13}C NMR data of **7b** (600.23 MHz for ^{1}H, 150.94 MHz for ^{13}C, D_2O, 30 °C).

	assignment	δ_C	m.	δ_H	n_H	m.	*J*[Hz]	HMBC (C to H)
**Boc**	**CO**	158.29	S	-	0			
	**C**	81.2[a]	S	-	0			$(CH_3)_3$
	**$(CH_3)_3$**	27.78	Q	1.250	9	s		
**spacer**	**1'**	44.3[a]	T	3.48[b]	2	m		
	**2'**	39.3[a]	T	3.083	2	m		
	**CS**	n.d.[c]	-	-	0			
**Glc^A^**	**1**	n.d.[c]	-	n.d.	-			
	**2**	54.09	D	3.74[b]	1	m		
	**3**	72.7[a]	D	3.63[b]	1	m		
	**4**	78.22	D	3.55[b]	1	m		**1^B^**
	**5**	76.12	D	3.49[b]	1	m		
	**6**	60.03	T	3.77[b]	1	m		4
				3.65[b]	1	m		
	**2-Ac**	22.12	Q	1.833	3	s		
	**CO**	175.10	S	-	0			2-Ac
**Gal^B^**	**1**	103.00	D	4.292	1	d	7.9	2, 3
	**2**	69.97	D	3.419	1	dd	10.9, 7.9	1, 3, 4
	**3**	82.08	D	3.53[b]	1	m		1, 2, 4, **1^C^**
	**4**	68.43	D	3.966	1	d	2.9	3
	**5**	75.09	D	3.54[b]	1	m		
	**6**	61.03	T	3.57[b]	2	m		4, 5
**Glc^C^**	**1**	102.83	D	4.51[b]	1	m		2, **3^B^**
	**2**	55.80	D	3.60[b]	1	m		3
	**3**	71.80	D	3.50[b]	1	m		
	**4**	78.22	D	3.61[b]	1	m		**1^D^**
	**5**	74.63	D	3.38[b]	1	m		
	**6**	60.03	T	3.74[b]	1	m		4
				3.64[b]	1	m		
	**2-Ac**	22.27	Q	1.851	3	s		
	**CO**	175.01	S	-	0			2, 2-Ac
**Gal^D^**	**1**	100.38	D	5.086	1	d	5.1	2, 3, 6, **4^C^**
	**2**	70.07	D	3.752	1	dd	5.1, 4.7	1, 3, 4
	**3**	65.88	D	4.179	1	dd	4.7, 3.8	2, 4
	**4**	123.03	D	6.037	1	d	3.8	2, 3
	**5**	147.86	S	-	0			1, 3, 4
	**6**	189.83	D	9.026	1	s		5

[a]broad signal; [b]HSQC readout; [c]n.d.: not detected

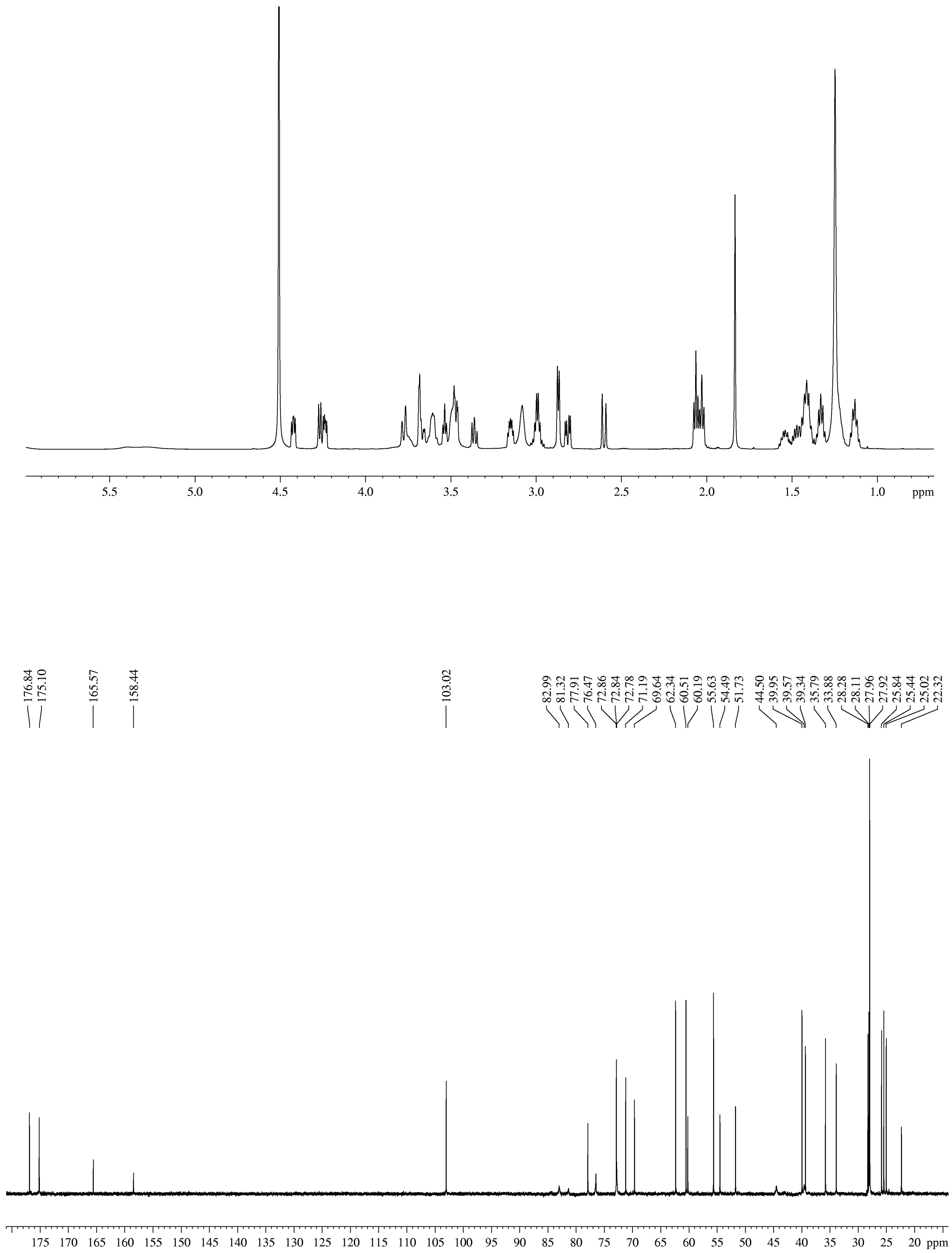

Figure NMR 5: ^{1}H and ^{13}C NMR spectra of **15a** (600.23 MHz for ^{1}H, 150.94 MHz for ^{13}C, D_2O, 30 °C).

Table NMR 5: ^{1}H and ^{13}C NMR data of **15a** (600.23 MHz for ^{1}H, 150.94 MHz for ^{13}C, D_2O, 30 °C).

	assignment	δ_C	m.	δ_H	n_H	m.	*J*[Hz]	HMBC (C to H)[d]
**Boc**	**CO**	158.44	S	-	0			2' w
	**C**	81.32	S	-	0			$(CH_3)_3$
	**$(CH_3)_3$**	27.96	Q	1.247	9	s		$(CH_3)_3$
**spacer**	**1'**	44.50	T	3.48[a]	2	m		
	**2'**	39.57	T	3.081	2	m		
	**CS**	183.1[b]	S	-	0			
**Glc[A]**	**1**	82.99	D	5.40, 5.28[a]	1	br s		
	**2**	54.49	D	3.75[a]	1	m		2-Ac
	**3**	72.78	D	3.61[a]	1	m		
	**4**	77.91	D	3.60[a]	1	m		5, 6d, **1[B]**
	**5**	76.47	D	3.49[a]	1	m		6d
	**6**	60.19	T	3.776	1	dd	12.4, 1.5	
				3.668	1	dd	12.4, 4.4	
	**2-CO**	175.10	S	-	0			2-Ac
	**2-Ac**	22.32	Q	1.834	3	s		
**Gal[B]**	**1**	103.22	D	4.269	1	d	7.8	2, 3, 5, **4[A]**
	**2**	71.19	D	3.361	1	dd	10.0, 7.8	1, 3, 4
	**3**	72.84[c]	D	3.471	1	dd	10.0, 3.3	
	**4**	69.64	D	3.685	1	d	3.3	3, 6
	**5**	72.86[c]	D	3.581	1	t	6.2	
	**6**	51.73	T	2.870	2	d	6.2	4, 5
**spacer**	**1"**	175.10	S	-	0			2", 3"
	**2"**	33.88	T	2.028	2	t		3", 4"
	**3"**	25.02	T	1.416	2	m		2", 4", 5"
	**4"**	25.84	T	1.131	2	m		2", 3", 5", 6"
	**5"**	28.28	T	1.331	2	m		6"
	**6"**	39.34	T	3.011	1	dt	13.5, 6.8	**2**, 4", 5"
				2.979	1	dt	13.5, 6.8	
**biotin**	**1**	176.84	S	-	0			**6"**, 2, 3u, 3d
	**2**	35.79	T	2.063	2	m		3u, 3d, 4
	**3**	25.44	T	1.46[a]	2	m		2, 4, 5u, 5d
	**4**	28.11	T	1.230	2	m		2
	**5**	27.95	T	1.547	1	m		6, 7, 9u
				1.404	1	m		
	**6**	55.63	D	3.150	1	m		4, 5u, 5d, 7, 8,
	**7**	62.34	D	4.238	1	dd	7.8, 4.5	5u, 5d, 8, 9u, 9d
	**8**	60.51	D	4.423	1	dd	7.8, 5.0	7, 9u
	**9**	39.95	T	2.815	1	dd	13.0, 5.0	7, 8
				2.602	1	d	13.0	
	**$CO(NH)_2$**	165.57	S	-	0			7, 8

[a]HSQC readout; [b]broad signal; [c]might be interchanged; [d]u: up-field, d: down-field

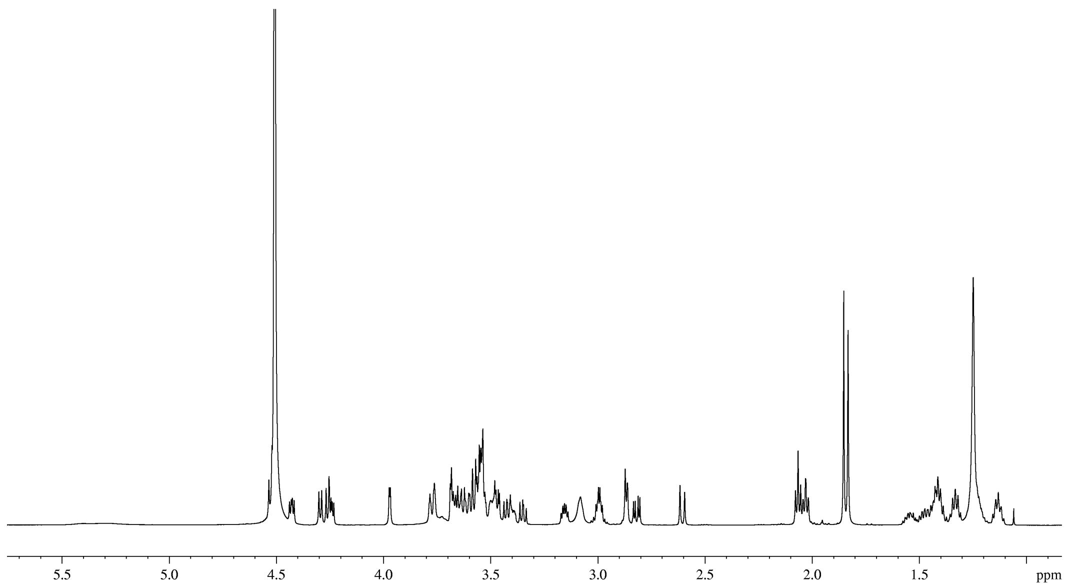

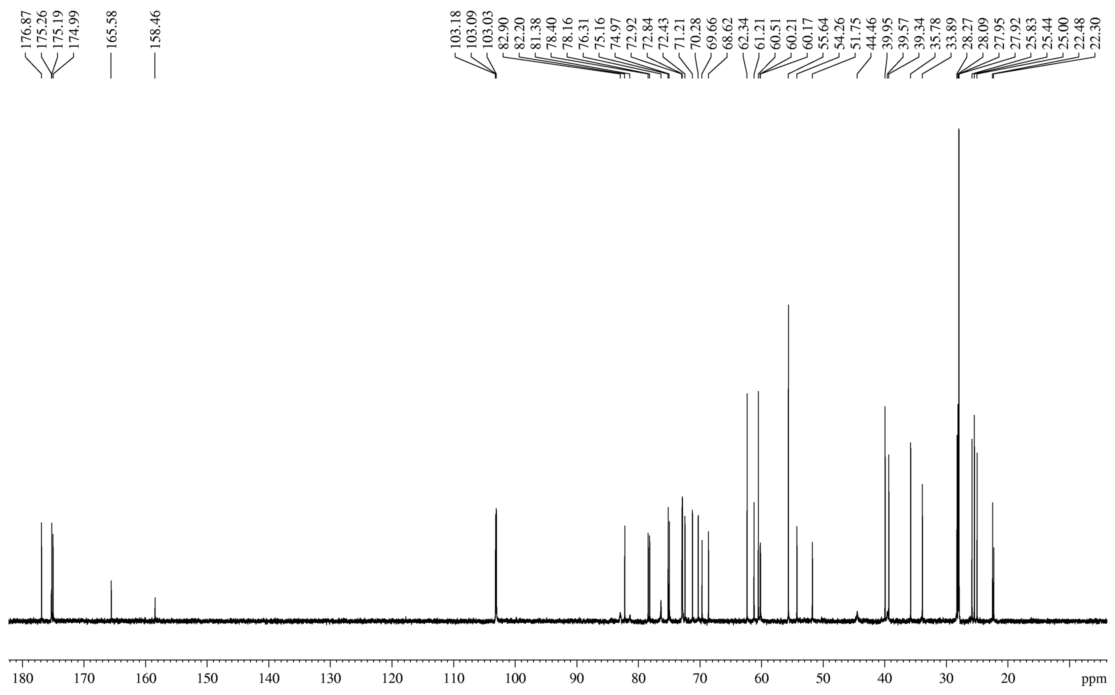

Figure NMR 6: ^{1}H and ^{13}C NMR spectra of **15b** (600.23 MHz for ^{1}H, 150.94 MHz for ^{13}C, D_2O, 30 °C).

Table NMR 6: ^{1}H and ^{13}C NMR data of **15b** (600.23 MHz for ^{1}H, 150.94 MHz for ^{13}C, D_2O, 30 °C).

	assignment	δ_C	m.	δ_H	n_H	m.	*J*[Hz]	HMBC (C to H)[c]
**Boc**	**CO**	158.46	S	-	0			2‘ w
	**C**	81.38	S	-	0			$(CH_3)_3$
	**$(CH_3)_3$**	27.95	Q	1.249	9	s		$(CH_3)_3$
**spacer**	**1‘**	44.46	T	3.483	2	m		
	**2‘**	39.57	T	3.082	2	m		
	**CS**	183.1[b]	S	-	0			
**Glc[A]**	**1**	82.90	D	5.29, 5.41[a]	1	br s		
	**2**	54.26	D	3.73[a]	1	m		4 w, 2-Ac
	**3**	72.88	D	3.62[a]	1	m		
	**4**	78.40	D	3.55[a]	1	m		**1[B]**
	**5**	76.31	D	3.50[a]	1	m		4, 6d
	**6**	60.21	T	3.77[a]	1	m		
				3.66[a]	1	m		
	**2-CO**	175.26	S	-	0			2-Ac
	**2-Ac**	22.30	Q	1.833	3	s		
**Gal[B]**	**1**	103.19	D	4.296	1	d	7.9	2, **4[A]**
	**2**	70.28	D	3.424	1	dd	9.9, 7.9	1, 3, 4
	**3**	82.20	D	3.547	1	m		1, 2, 4, **1[C]**
	**4**	68.62	D	3.972	1	d	3.3	3
	**5**	75.16	D	3.542	1	m		1, 4, 6
	**6**	61.21	T	3.569	2	m		4, 5
**Glc[C]**	**1**	103.03	D	4.529	1	d	8.5	2, **3[B]**
	**2**	55.64	D	3.638	1	dd	9.9, 8.5	1, 3
	**3**	72.43	D	3.537	1	dd	9.9, 9.5	1, 2, 4
	**4**	78.16	D	3.585	1	m		3, 6d, **1[D]**
	**5**	74.94	D	3.401	1	ddd	9.7, 4.6,	1, 4, 6u, 6d
	**6**	60.17	T	3.776	1	m		4
				3.670	1	m		
	**2-CO**	174.99	S	-	0			2-Ac
	**2-Ac**	22.48	Q	1.853	3	s		
**Gal[D]**	**1**	103.09	D	4.262	1	d	7.9	2, **4[C]**
	**2**	71.21	D	3.349	1	dd	9.9, 7.9	1, 3, 4
	**3**	72.84	D	3.471	1	dd	9.9, 3.4	1, 2
	**4**	69.66	D	3.686	1	m		3, 5, 6
	**5**	72.92	D	3.535	1	m		6
	**6**	51.75	T	2.868	2	d	6.2	4, 5
**spacer**	**1”**	175.19	S	-	0			2”, 3”
	**2”**	33.89	T	2.032	2	m		3”, 4”
	**3”**	25.00	T	1.415	2	m		2”, 4”, 5”
	**4”**	25.83	T	1.132	2	m		2”, 3”, 5”, 6”
	**5”**	28.27	T	1.333	2	m		4”,6”
	**6”**	39.34	T	3.009	1	dt	13.5, 6.8	4”, 5”, **2**
				2.908	1	dt	13.5, 6.8	

**biotin**	**1**	176.87	S	-	0			2, 3, **6”**
	**2**	35.78	T	2.066	2	m		3, 4
	**3**	25.44	T	1.46[a]	2	m		2, 4, 5u, 5d
	**4**	28.09	T	1.231	2	m		2, 6
	**5**	27.92	T	1.547	1	m		6, 7 w, 9u w
				1.405	1	m		
	**6**	55.64	D	3.156	1	m		4, 5u, 5d, 7, 8,
	**7**	62.34	D	4.243	1	dd	7.9, 4.6	5u, 5d, 8, 9u
	**8**	60.51	D	4.429	1	ddd	7.9, 5.0,	7, 9u
	**9**	39.95	T	2.819	1	dd	13.0, 5.0	7, 8
				2.607	1	d	13.0	
	**CO(NH)$_2$**	165.58	S	-	0			7, 8, 9u

[a]HSQC readout; [b]broad signal; [c]w: weak signal, u: up-field, d: down-field

Table NMR 7: ^{15}N NMR data of **15b** (600.23 MHz for ^{1}H, 60.82 MHz for ^{15}N, D_2O, 30 °C).

assignment	δ_N[a]	HMBC (N to H)
2-**N**HAc^{A}	121.0	2-Ac^{A}, 3^{A}
2-**N**HAc^{C}	121.0	2-Ac^{C}, 3^{C}
6^{D}-**N**H	65.2	5^{D}, 6^{D}
1”-**N**H	146.6	6^{D}, 2”
6”-**N**H	125.6	6”, b2
b7-**N**H	90.7	b7, b9-u, b9-d
b8-**N**H	81.9	b6, b8

[a]^{1}H,^{15}N HMBC readouts
